# Effect of a digitally augmented general health promotion intervention on abstinence from health-risk behaviors among emergency department discharge patients: A randomized controlled trial

**DOI:** 10.1371/journal.pmed.1004916

**Published:** 2026-09-08

**Authors:** Ho Cheung William Li, Wei Xia, Joanna Yeung, Laurie Long Kwan Ho, Tan Ankie Cheung, Kai Yeung Cheung, Yiu Cheung Chan, Wai Yee Chan, Chin Wai Lui, Hong Chen

**Affiliations:** 1 The Nethersole School of Nursing, The Chinese University of Hong Kong, Shatin, Hong Kong SAR, China; 2 School of Nursing, Sun Yat-Sen University, Guangzhou, China; 3 United Christian Hospital, Kowloon, Hong Kong SAR, China; University of North Carolina, UNITED STATES OF AMERICA

## Abstract

**Background:**

Noncommunicable diseases (NCDs) are the leading global cause of death and are driven by modifiable behaviors, such as tobacco use, harmful alcohol consumption, unhealthy diet, and physical inactivity. Recognizing that emergency department (ED) visits represent a unique opportunity to promote behavior change, this trial evaluated a digitally augmented, theory based general health promotion approach, combining a brief telephone-based intervention with mobile instant messaging support, to help discharged ED patients abstain from health risk behaviors.

**Methods and findings:**

This assessor-blinded randomized controlled trial was conducted in a major public hospital ED in Hong Kong. Adults (18–65 years) triaged as semi-urgent or non-urgent and with ≥1 health-risk behavior and smartphone access were randomized to receive a digitally augmented, theory‑based general health‑promotion intervention consisting of a brief telephone‑based AWARD‑model intervention (Ask, Warn, Advise, Refer, and Do-it-again) followed by weekly WhatsApp or WeChat messages for 6 months, or to a control group receiving brief telephone advice only. The primary outcome was self-report abstinence from ≥1 health-risk behavior at 6 months; secondary outcomes included the proportion of participants who achieved self-reported abstinence from ≥1 health-risk behavior at 12 months and reduction in the number of behaviors at 6 and 12 months. Of the 2,134 screened patients, 572 were enrolled (286 per group). At 6 months, 30.1% of the intervention participants versus 19.9% of the controls achieved self-reported abstinence (RR = 1.51; 95% CI, 1.13–2.02; *P* = 0.006). The intervention also significantly increased the likelihood of fewer risky behaviors at 6 (RR = 1.54; *P* = 0.01) and 12 (RR = 1.48; *P* = 0.02) months. Physical inactivity showed the greatest improvement at 6 months (31.7% versus 16.2%; *P* < 0.001). The effects attenuated after cessation of booster messaging. Limitations include reliance on self-reported outcomes, the single-center study design, and loss to follow-up, which may have affected the generalizability of the results.

**Conclusions:**

A digitally augmented, theory-based general health promotion strategy delivered at ED discharge through brief telephone intervention and mobile instant messaging support demonstrated short-term benefits in promoting self-reported abstinence and reducing health-risk behaviors at 6 months. However, the absence of a sustained effect at 12 months suggests that extended support or maintenance strategies may be required to maintain these improvements over time. Multicenter trials with longer follow-up are warranted to evaluate long-term effectiveness.

**Clinical trial registration:**

ClinicalTrials.gov (Registration No: NCT06077565).

## Author summary

**Why was this study done?:**

- Noncommunicable diseases such as heart disease, diabetes, cancer, and chronic lung disease are responsible for most deaths worldwide and are strongly linked to unhealthy behaviors, including smoking, harmful alcohol use, poor diet, and lack of physical activity.
- People who visit an emergency department (ED) may be particularly open to advice about improving their health because they are seeking care during an episode of illness or discomfort.
- We wanted to test whether a simple health-promotion program combining telephone advice and mobile messaging could help patients reduce unhealthy behaviors after discharge from the ED.

**What did the researchers do and find?:**

- We conducted a randomized controlled trial involving 572 adults attending a public hospital ED in Hong Kong who reported at least one health-risk behavior.
- Participants received either a brief telephone-based health-promotion intervention followed by weekly WhatsApp or WeChat messages for 6 months, or brief advice alone.
- After 6 months, participants receiving the intervention were more likely to stop at least one health-risk behavior (30.1% versus 19.9%) and were more likely to reduce their overall number of risk behaviors than those receiving brief advice alone.
- The largest improvement was seen in physical activity, but the benefits became weaker after the 6-month messaging support ended.

**What do these findings mean?:**

- A low-cost health-promotion program delivered after ED discharge can help people make positive lifestyle changes and reduce behaviors that increase their risk of chronic disease.
- Because the intervention uses brief counseling and commonly used mobile messaging platforms, it could potentially be incorporated into routine ED care and delivered to large numbers of patients.
- The benefits were mainly seen during the active intervention period, and the study was conducted in one hospital, relied on participants’ self-reports, and had some loss to follow-up, so further studies are needed to confirm whether the approach produces lasting effects in other settings.

## Abstract 3

Ho Cheung William Li and colleagues conduct a randomized controlled trial to assess the effects of a health-promotion intervention delivered by telephone, with weekly digital reminders and initiated at hospital discharge, that aims to improve abstinence from health risk behaviors.

## Introduction

Chronic diseases, also known as noncommunicable diseases (NCD), include cardiovascular diseases, cancers, chronic respiratory diseases, and diabetes [1]. Together, NCDs have become the leading causes of morbidity and mortality worldwide and now account for more than 70% of all deaths [1]. The WHO has identified tobacco use, harmful alcohol consumption, unhealthy diet, and physical inactivity as the major behavioral risk factors that significantly contribute to NCDs and increase the risk of premature death [2]. Hypertension, in particular, is one of the most prevalent and frequently underdiagnosed NCDs globally and is a major contributor to cardiovascular morbidity commonly encountered in emergency department (ED) settings [3]. In Hong Kong, the burden of NCDs is growing, intensified by an aging population [4]. The Hong Kong Hospital Authority projects a 50% increase in NCD cases among middle-aged individuals, with the 2 million cases reported in 2019 expected to rise to 3 million by 2039, thereby creating a serious challenge to the healthcare system and adding to the long-term socioeconomic burden [5].

Most premature deaths from NCDs are preventable through lifestyle modifications [6]. Therefore, supporting individuals’ efforts to abstain from health-risk behaviors, such as quitting smoking, avoiding harmful alcohol consumption, maintaining a balanced diet, and engaging in regular physical activity, can help prevent or control high-prevalence conditions such as hypertension, cardiovascular disease, and diabetes, and improve overall population health [7]. Smoking, in particular, is a well‑established risk factor for hypertension and cardiovascular disease, underscoring the importance of helping smokers quit smoking as part of a broader NCD prevention strategy [3]. However, many individuals struggle with motivation or find abstaining from health-risk behaviors to be challenging, particularly when they receive minimal guidance and support from healthcare professionals [8–11]. Consequently, ED visits present a valuable opportunity for encouraging positive health behavior changes, as individuals seeking care during episodes of physical discomfort may be more receptive to advice on reducing health-risk behaviors.

According to the Hospital Authority Annual Report [12], Hong Kong hospitals reported 1.8 million ED visits between 2021 and 2022, with over half of the cases triaged as semi-urgent (level 4) or non-urgent (level 5). Most of these patients were discharged home after receiving medical attention. Conditions commonly associated with NCDs, including poorly controlled hypertension and other cardiovascular‑related complaints, account for a substantial proportion of ED attendances in this group. This highlights the ED as a strategic setting to address prevalent NCD risk factors and provide timely health advice prior to discharge to encourage abstinence from health‑risk behaviors.

Previous studies have shown that individuals who have a general intention to improve their health are more likely to adopt desirable health-related lifestyles, and that once engaged, these individuals also tend to progress toward abstaining from health-risk behaviors [13, 14]. Based on this concept and previous supporting results, we conducted a pilot study to evaluate a general health promotion approach delivered via instant messaging (WhatsApp or WeChat) aimed at helping smokers quit smoking [15]. The patients in this pilot study who had medical follow-up in a special outpatient clinic ultimately quit smoking, and more than 90% of the participants owned a smartphone and could use an instant messaging application [15]. The participants were encouraged to select and abstain from a health-risk behavior (e.g., tobacco use, harmful alcohol consumption, unhealthy eating, or physical inactivity). The pilot study findings confirmed the feasibility and potential benefits of using a general health-promotion approach to motivate smokers who initially had no intention of quitting to eventually take action to quit [15].

Based on the pilot study findings, the present study explored whether robust evidence for the efficacy of this approach could be obtained in a full-scale randomized controlled trial (RCT) aimed at motivating ED patients to abstain from health-risk behaviors upon discharge. We hypothesized that, compared with a nonintervention control group, participants receiving the intervention using the general health-promotion approach would achieve significantly greater success in abstaining from health-risk behaviors at 6- and 12-month follow-ups after ED discharge.

## Methods

### Study design

This assessor-blinded RCT used a two-group parallel design. The study was prospectively registered before enrollment of the first participant in ClinicalTrials.gov, a WHO-recognized clinical trial registry (registration number NCT06077565; https://clinicaltrials.gov/study/NCT06077565). The trial was conducted and reported in accordance with the CONSORT 2025 guidelines (S1 Checklist), and the study protocol is available in S1 Text. The intervention was designed based on principles from the Theory of Planned Behavior [16], the foot-in-the-door technique [17], and Self-Determination Theory [18–20]. Details of the theoretical framework are provided in the Supplementary Information (S2 Text).

### Ethics statement

Ethical approval was granted by the Research Ethics Committee (Kowloon Central/ Kowloon East) [REC(KC/KE)] with the reference number KC/KE-23-0164/FR-3. The study was conducted in accordance with the Declaration of Helsinki (https://www.wma.net/what-we-do/medical-ethics/declaration-of-helsinki/) and adhered to all relevant ethical principles for the design and conduct of clinical research. Written informed consent was obtained from all eligible participants after the study aims were explained.

### Participants and settings

Chinese patients who attended the ED of a major acute care public hospital in Hong Kong because of physical discomfort and who fulfilled the following criteria were invited to participate. Inclusion criteria included (1) 18–65 years, (2) triage as semi-urgent (level 4) or nonurgent (level 5) with expected discharge home the same day after seeking medical attention, (3) the presence of at least one health risk behavior (tobacco use, harmful alcohol consumption, unhealthy diet, or physical inactivity), and (4) able to communicate in Chinese. Exclusion criteria included (1) poor cognitive state or mental illness, (2) diagnosis with NCDs with regular specialist outpatient follow‑up, defined as scheduled specialist clinic visits at least once every 3–6 months over the preceding year; and (3) participation in another related study.

The criteria for determining each health risk behavior are listed in S1 Table. In the Hong Kong context, smartphone ownership and use of instant messaging applications are near‑universal; according to the 2023 Thematic Household Survey [21], 96.4% of residents own a smartphone and regularly use instant messaging applications. Individuals who did not have a smartphone or who did not use WhatsApp/WeChat (unlikely in Hong Kong) were provided brief advice and a Practical Resource Hub for Healthy Life leaflet on healthy lifestyles but were otherwise excluded from the study.

All patients were approached by emergency nurses prior to discharge from the ED. The emergency nurse provided potential participants with an information leaflet outlining the study’s nature and objectives. Interested individuals were then referred to a research assistant for further discussion. Participants indicated their willingness to take part in the study by signing an informed consent form. They were assured that participation was entirely voluntary, that refusal would not affect their care, and that all information provided would remain confidential.

### Sample size

G*Power was used to estimate the sample size based on the results of our pilot study [15]. The trial was powered to evaluate the overall effectiveness of a theoretically informed intervention package, rather than to test individual theoretical constructs or mediational pathways.

At the time of study design, no closely comparable randomized trials targeting multiple health risk behaviors in emergency department settings were available to inform the calculation. The pilot study demonstrated an approximately 10% between-group difference in sustained abstinence from at least one health-risk behavior at the 12-month follow-up (16.7% in the intervention group versus 6.7% in the control group), assessed using a biochemically validated quit rate.

For sample size estimation, no closely comparable studies were available at the time of study design; therefore, estimates were informed by data from our pilot study. For the purposes of power calculation, we used the biochemically validated quit rate at 12 months from the pilot study, as this represented the most objective and conservative outcome measure available. The calculation assumed a two-sided test of two independent proportions based on a normal approximation (Z-test), with a type I error rate of 5% (*α* = 0.05, two-tailed) and 80% power. Under these assumptions, 199 participants per group were required to detect the observed between-group difference. After allowing for an anticipated 30% attrition rate at the 12-month follow-up, the total target sample size was increased to 573 participants.

The primary outcome was abstinence from at least one health-risk behavior at 6 months. Although self-reported behavioral change at 12 months was originally specified as the primary outcome in the trial registration, this was revised before trial commencement, prior to participant recruitment and data collection, following internal discussion and recommendations received during the funding review process. Accordingly, the sample size calculation was based on what was originally the registered primary outcome (12-month abstinence), which was subsequently retained as a secondary outcome before recruitment commenced. The sample size was not recalculated following this change because intervention effects were expected to be greatest at the end of the active 6-month intervention period and therefore were not anticipated to be smaller than those observed at 12 months. The modification reflected the design of the intervention, which provided active support and booster messaging over a 6-month period. As intervention effects were expected to be greatest at the completion of the active intervention phase, the 6-month assessment was considered the most appropriate primary endpoint for evaluating intervention efficacy. The 12-month assessment was retained as a secondary outcome to evaluate the sustainability of intervention effects beyond the intervention period. Consequently, the primary outcome reported in this manuscript differs from that originally specified in the trial registration.

In total, 572 participants were recruited and randomized in a 1:1 ratio to the intervention and control groups. The primary analysis was conducted according to the intention-to-treat principle, whereby all randomized participants were retained in the analysis regardless of follow-up completion status.

### Randomization and allocation concealment

To avoid the potential risk of treatment contamination within the ED, randomization was not performed there. Instead, the research assistant inputted the baseline data collected at the ED directly into the web-based trial entry form linked to a computerized database, and randomization was performed at the principal investigator’s institution by an independent statistician who had no other involvement in the study. Stratified block randomization with 1:1 allocation was conducted using varying block sizes of 4–8 to achieve an appropriate balance of participant numbers between the two groups and to optimize allocation concealment. Randomization was stratified by health-risk behaviors (tobacco use, harmful alcohol consumption, unhealthy diet, and physical inactivity) to yield a balanced allocation of participants with different lifestyle risk profiles between the two groups.

### Intervention

A baseline assessment was performed using questionnaires at the ED by a research assistant before the patients were discharged home. All participants were informed that they would receive a telephone call from a research assistant within 3 days to evaluate their potential health-risk behaviors and provide them with appropriate health advice to support them to abstain from health-risk behaviors. The participants were also given a Practical Resource Hub for Healthy Life leaflet (https://www.chp.gov.hk/files/pdf/qr_code_healthy_and_joyful_tools_bi.pdf) containing information on various applications, including (i) “Move Your Body,” (ii) “Eat Healthy,” (iii) “Live Alcohol Free,” and (iv) “Stay Away from Tobacco,” which were developed by the Hong Kong Department of Health.

#### Intervention group.

***Brief intervention:*** The participants received a brief intervention via telephone (within 3 days after visiting the ED), conducted by a research nurse using the AWARD model (Ask, Warn, Advise, Refer, and Do-it-again). The intervention was originally developed for tobacco cessation in primary care patients [9,10,22].

In the present study, the AWARD model was minimally adapted to support a general health-promotion approach while retaining its original five steps: (1) ask about and assess health-risk behaviors; (2) warn about the high morbidity and mortality risks associated with health-risk behaviors; (3) advise to abstain from health-risk behaviors; (4) refer to hotline services on request, such as smoking cessation and alcohol treatment services; and (5) repeat steps 1–4 again if participants failed to abstain from health-risk behavior at follow-ups.

The AWARD model functions as a structured counseling framework rather than an independent theoretical model. In this study, AWARD served as the delivery structure for a theory‑informed intervention. The “Ask” and “Warn” components align with the Theory of Planned Behavior by targeting attitudes and risk perception; the “Advise” and “Refer” steps were delivered in an autonomy‑supportive manner consistent with self‑determination theory; and the “Do‑it‑again” component reflects the foot‑in‑the‑door principle through iterative, progressive engagement.

The core structure, counseling sequence, and intensity of the AWARD model were unchanged. The sole adaptation involved broadening the behavioral focus from smoking to multiple health risk behaviors, including tobacco use, harmful alcohol consumption, unhealthy diet, and physical inactivity. This modified application has been previously reported in published randomized controlled trial protocol [23] and was implemented as a pragmatic extension of an established intervention framework rather than as a newly developed or revalidated model.

For the warning message, the research assistant provided the following standardized message: “The WHO has identified four major behavioral risk factors, namely, tobacco use, harmful alcohol consumption, an unhealthy diet, and physical inactivity, that contribute substantially to NCDs, which result in high rates of morbidity and mortality.” For the advice step, the research nurse asked participants to first select and abstain from a health-risk behavior that they considered easiest to achieve, such as tobacco use, harmful alcohol consumption, unhealthy eating, or physical inactivity. The participants were encouraged to abstain from health-risk behaviors sequentially, but they were also allowed to choose to quit them simultaneously if they were confident in their ability to do so. Each participant then received a brief (approximately 5 min) individual intervention with health advice about the selected health-risk behavior. The whole intervention lasted approximately 10 min (slightly longer, if necessary). At the end of the telephone call, the participants were informed that, throughout the study period, the research nurse would assist in achieving the participant’s health-related goals by communicating via WhatsApp or WeChat messages or telephone calls.

***Follow-up booster intervention (up to 6 months):*** During the first 6 months of the study period, the research nurse sent WhatsApp or WeChat messages approximately once per week to remind participants to abstain from the selected health-risk behavior. Instant messaging via mobile applications has previously been confirmed to enhance treatment compliance [24]. The weekly WhatsApp or WeChat messages were standardized, with all participants receiving the same pre-defined message content. The messages were one way and non-interactive; no response from participants was required or solicited. Messages were delivered weekly for up to 6 months following baseline assessment. The average number of messages delivered was 24 per participant (range 22–26), reflecting minor variations due to scheduling and public holidays. The content of the weekly digital messages is provided in S3 Text.

***Follow-up assessment of behavioral changes at 3, 6, and 12 months:*** Follow‑up assessments were conducted at 3, 6, and 12 months after enrollment via telephone interviews. Outcome assessments were carried out by research nurses who were not involved in delivering the initial intervention or booster messaging and who followed a standardized assessment script.

During assessments, participants were asked structured questions regarding their current health‑risk behaviors. For participants in the intervention group, if abstinence from the initially selected health‑risk behavior was reported, the research nurse encouraged the participant to consider abstaining from an additional health‑risk behavior, if present, and provided brief health counseling (approximately 5 min) focused on the newly selected behavior. This counseling formed part of the intervention protocol and was delivered only to participants allocated to the intervention group.

#### Control group.

Participants allocated to the control group received a single brief telephone intervention within three days of ED discharge, delivered by a research nurse using a simplified AWARD‑based approach. Similar to the intervention group, this call addressed the participant’s health‑risk behaviors; however, during the advice step, participants were only asked to consider abstaining from their identified health‑risk behaviors with reference to the Practical Resource Hub for Healthy Life leaflet. No structured counseling, individualized goals, or booster support were provided. Participants in the control group did not receive any WhatsApp or WeChat messages or additional behavioral counseling after this initial call. Follow‑up contacts at 3, 6, and 12 months were conducted solely for outcome assessment purposes, following the same assessment schedule as the intervention group, without behavioral encouragement, reinforcement, or intervention content.

### Measures

The primary outcome was the proportion of participants who achieved abstinence from at least one health-risk behavior at 6 months. Secondary outcomes included the proportion of participants who achieved abstinence from at least one health-risk behavior at 12 months and a reduced number of behaviors at 6 and 12 months. The criteria for assessing each health-risk behavior and defining successful abstinence, including tobacco use [25], binge drinking [13], unhealthy diet [26], and insufficient physical activity [27], are presented in S1 Table.

Although quality-of-life and cost-effectiveness outcomes were initially proposed in the funding application and early trial planning documents, they were not included in the final ethics-approved protocol prior to trial commencement and were therefore not measured. This decision was made following methodological and statistical consultation, which concluded that the study duration was insufficient to support a robust assessment of longer-term quality-of-life changes and cost-effectiveness. Consequently, these outcomes were removed from the final protocol before participant recruitment commenced.

A behavioral risk-factor survey was used to collect the eligible participants’ demographic and clinical data and information about their health-risk behaviors at baseline and at 3, 6, and 12 months. The behavioral risk factor survey items were derived from standardized public health surveillance tools used by the Hong Kong Department of Health and the World Health Organization. These items assess observable behaviors with clear face validity; although they have not undergone formal psychometric validation, they are widely used in local and international population health surveys.

The demographic data included age, gender, socioeconomic status, and clinical characteristics. This questionnaire was adapted from a questionnaire used by the Hong Kong Department of Health to investigate multiple health-risk behaviors among Chinese adults in Hong Kong in 2021 [28].

#### Safety monitoring.

Safety was monitored throughout the study by the research team. No study-related adverse events were identified or reported.

### Statistical methods

SPSS for Windows (SPSS version 26.0; IBM Corp., Armonk, NY, USA) was used for the quantitative data analysis. Descriptive statistics were used to calculate the mean, standard deviation, and frequency of the demographic and health-risk behavior data. The baseline characteristics of the two groups were compared using the chi-squared test for categorical variables and *t* test for continuous variables. An intention-to-treat (ITT) analysis was used by imputing all non-responses at follow-up by baseline values (i.e., assuming failure or no change after the intervention) to yield more conservative effect size estimates.

The primary analysis was performed using a chi-squared test to assess the main effect by comparing the proportions of participants in the intervention and control groups who abstained from at least one type of health-risk behavior at 6 months. Secondary outcomes were analyzed using the same approach. Generalized estimating equation (GEE) models were employed to calculate the relative risks (RRs) for (1) abstaining from at least one type of health-risk behaviors, and (2) reducing the total number of health-risk behaviors at 6 and 12 months in the intervention group versus control group, using crude and adjusted models that accounted for baseline differences. Similarly, chi-squared test (or Fisher’s exact test when any cell had ≤ 5 participants) and GEE models were used to assess the differences in proportions of participants who abstained from any health-risk behavior at 6 and 12 months.

To assess the robustness of the primary ITT analysis, a post-hoc complete case analysis (CCA) was conducted as a sensitivity analysis, excluding participants with missing outcome data at each follow up time point. We conducted a sensitivity analysis additionally adjusting for baseline diet, physical activity, alcohol use, and smoking status. To assess the impact of missing outcome data, we performed multiple imputations using chained equations with 40 imputations, stratified by treatment group. The imputation model included all covariates from the primary analysis as well as variables associated with missingness. The primary analysis was then repeated across the imputed datasets using a modified Poisson regression model with robust standard errors.

#### Artificial intelligence (AI) tools and technologies.

No AI tools or technologies were used in the design, conduct, analysis, interpretation, or reporting of this study. All analyses and manuscript preparation were performed by the authors, who retain full responsibility for the final manuscript and all supporting materials.

## Results

**Fig 1:**
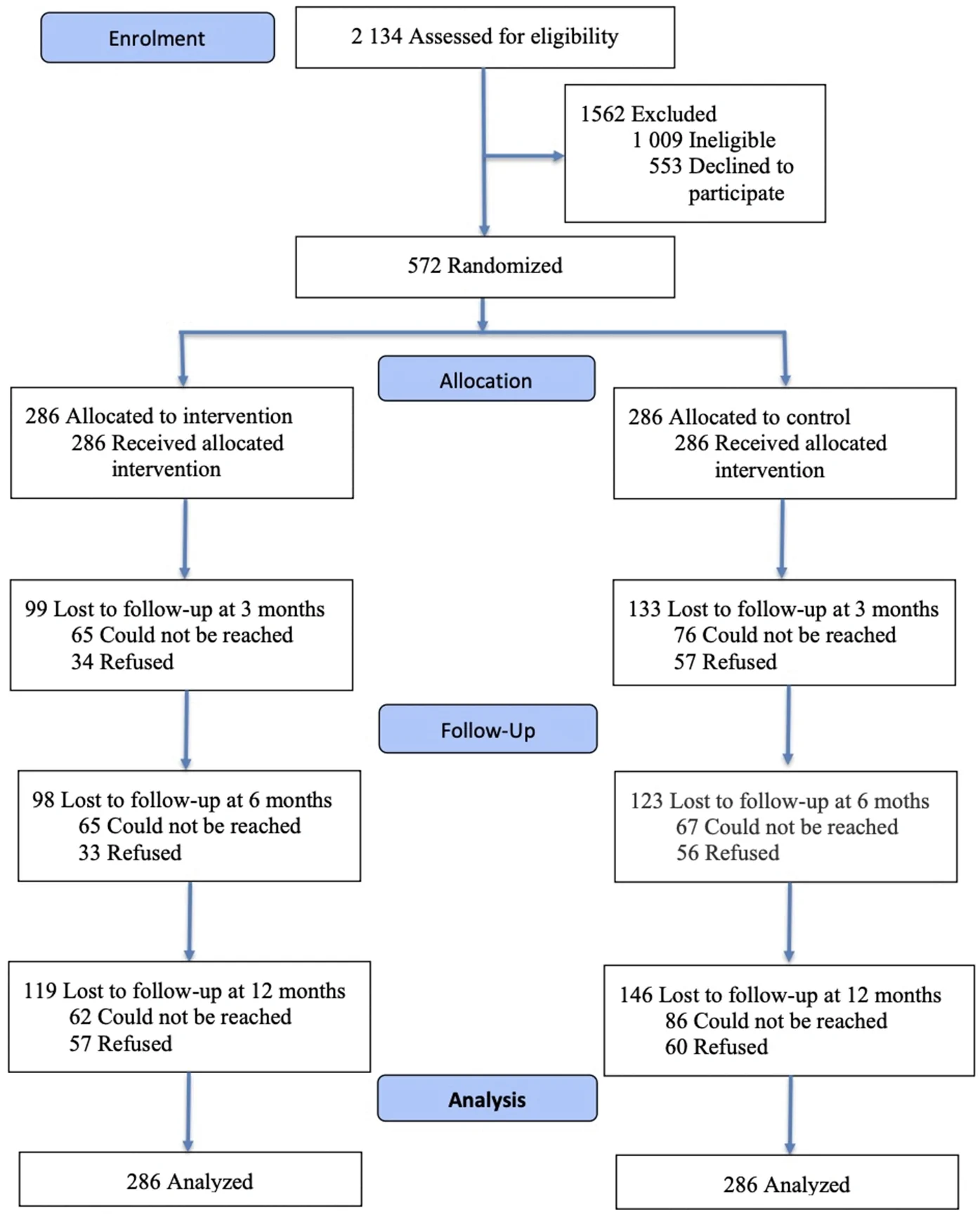
CONSORT flow diagram of participant screening, enrollment, randomization, follow-up, and analysis. Of 2,134 patients assessed for eligibility, 572 were randomized to the intervention (*n* = 286) or control group (*n* = 286). All randomized participants were included in the intention-to-treat analysis.

Patient enrollment took place between 15 January 2024 (first participant enrolled) and 15 April 2024 (last participant enrolled). During the data collection, we approached 2,134 patients presenting to the ED and identified 1,125 eligible participants, yielding an eligibility rate of 52.7%. Of these, 572 patients provided consent and were randomized into either the intervention group (*n* = 286) or the control group (*n* = 286), resulting in a response rate of 50.8% (Fig 1). Follow‑up completion rates at 3, 6, and 12 months were 65.4%, 65.7%, and 58.4% in the intervention group, and 53.5%, 57.0%, and 49.0% in the control group, respectively, with significantly higher completion rates in the intervention group at all follow‑up time points (all *p* < 0.05).

Table 1 summarizes the demographic data of the participants. The majority were female (53.3%), with a mean age of 42.2 ± 12.8 years. Over half were married (55.8%) and employed (69.6%), and most (73.6%) had completed upper secondary education or higher. Baseline characteristics were generally comparable between groups. However, a higher proportion of males was observed in the control group, which likely contributed to the higher prevalence of binge drinking in this group.

**Table 1:** Participants’ demographic and health-risk behaviors at baseline (*N* = 572).

Variables	*N* (%)		
	Total	Control (*n* = 286)	Intervention (*n* = 286)
Age, mean (SD), years	42.2 (12.8)	42.0 (13.0)	42.4 (12.5)
Sex			
Male	267 (46.7)	145 (50.7)	122 (42.7)
Female	305 (53.3)	141 (49.3)	164 (57.3)
Education attainment			
Lower secondary or below	128 (22.4)	64 (22.4)	64 (22.4)
Upper secondary	206 (36.0)	105 (36.7)	101 (35.3)
Tertiary or above	215 (37.6)	107 (37.4)	108 (37.8)
Refused/Missing	23 (4.0)	10 (3.5)	13 (4.5)
Marital status			
Single/divorced/widowed	236 (41.3)	127 (44.4)	109 (38.1)
Married	319 (55.8)	153 (53.5)	166 (58.0)
Refused/Missing	17 (3.0)	6 (2.1)	11 (3.9)
Employment status			
Unemployed/Retired	174 (30.4)	88 (30.8)	86 (30.1)
Employed	398 (69.6)	198 (69.2)	200 (69.9)
Number of health-risk behaviors			
1	166 (29.0)	87 (30.4)	79 (27.6)
2	326 (57.0)	155 (54.2)	171 (59.8)
3	66 (11.5)	35 (12.2)	31 (10.8)
4	14 (2.5)	9 (3.2)	5 (1.8)
Smoking	89 (15.6)	45 (15.7)	44 (15.4)
Binge drinking	62 (10.8)	39 (13.6)	23 (8.0)
Unhealthy diet	515 (90.0)	256 (89.5)	259 (90.6)
Inadequate physical activity	406 (71.0)	198 (69.2)	208 (72.7)

^a^Calculated by *t* test or chi-squared test.

At baseline, 29.0%, 57.0%, 11.5%, and 2.5% of participants exhibited one, two, three, and four health-risk behaviors, respectively. Overall, 10.8% (62/572) reported binge drinking, 15.6% (89/572) reported smoking, 71.0% (406/572) reported physical inactivity, and 90.0% (515/572) reported an unhealthy diet.

Table 2 presents the clustering of the four health-risk behaviors. Among the participants reporting binge drinking, 98.4% (61 of 62) exhibited multiple health-risk behaviors, representing the highest proportion of clustering. Smoking was associated with the second-highest clustering, with 97.7% (87 of 89) of smokers engaging in more than one health-risk behavior.

**Table 2:** The clustering of four health-risk behaviors (*N* = 572).

	Health-risk behaviors, No./Total No. (%)			
	One	Two	Three	Four
Total	166/572 (29.0)	326/572 (57.0)	66/572 (11.5)	14/572 (2.5)
Smoking	2/89 (2.3)	26/89 (29.2)	47/89 (52.8)	14/89 (15.7)
Binge drinking	1/62 (1.6)	15/62 (24.2)	32/62 (51.6)	14/62 (22.6)
Unhealthy Diet	122/515 (23.7)	314/515 (61.0)	65/515 (12.6)	14/515 (2.7)
Inadequate physical activity	41/406 (10.1)	297/406 (73.1)	54/406 (13.3)	14/406 (3.5)

Table 3 summarizes the outcomes at the 6- and 12-month follow-ups. At 6 months, the proportion of participants abstaining from at least one health-risk behavior was significantly higher in the intervention group (86 of 286 [30.1%]) than in the control group (57 of 286 [19.9%]), corresponding to a risk ratio (RR) of 1.51 (95% CI, 1.13–2.02; *P* = 0.006). At 12 months, the intervention group continued to show a higher proportion of abstinence (61 [21.3%] versus 49 [17.1%]), although the difference was not statistically significant (RR, 1.24; 95% CI, 0.89–1.75; *P* = 0.205).

**Table 3:** Generalized equation model for primary and secondary outcomes at 6- and 12-month follow-up among 572 participants.

	No./Total No. (%)				RR (95% CI)^b^		aRR (95% CI)^b, c^	
	Total	Control	Intervention	*P* ^a^	Control	Intervention	Control	Intervention
**Primary outcome**								
Abstinence of ≥1 health-risk behavior at 6 months	143/572 (25.0)	57/286 (19.9)	86/286 (30.1)	**0.005**	1	1.51 (1.13–2.02)^**^	1	1.50 (1.12–2.01)^**^
**Secondary outcomes**								
Abstinence of ≥1 health-risk behavior at 12 months	110/572 (19.2)	49/286 (17.1)	61/286 (21.3)	0.20	1	1.24 (0.89–1.75)	1	1.20 (0.85–1.69)
Having reduced the total number of health-risk behaviors								
6 months	117/572 (20.5)	46/286 (16.1)	71/286 (24.8)	**0.01**	1	1.54 (1.11–2.15)^*^	1	1.51 (1.08–2.10)^*^
12 months	119/572 (20.8)	48/286 (16.8)	71/286 (24.8)	**0.02**	1	1.48 (1.07–2.05)^*^	1	1.45 (1.04–2.01)^*^

^a^Chi-squared test.

^b^Derived from a generalized estimating equation model with a binomial log link function using robust standard errors clustered by participant.

^c^Adjusted for age, sex, education level.

* *p* < 0.05; ** *p* < 0.01; *** *p* < 0.001.

For the reduction in the number of health-risk behaviors, reductions were achieved by a significantly greater proportion of participants in the intervention group than in the control group at both 6 months (71 [24.8%] versus 46 [16.1%]; RR, 1.54; 95% CI, 1.11–2.15; *P* = 0.011) and 12 months (71 [24.8%] versus 48 [16.8%]; RR, 1.48; 95% CI, 1.07–2.05; *P* = 0.019).

At the 6-month follow-up, after adjusting for age, gender, and education level, abstinence from at least one health-risk behavior was significantly more likely in the intervention group than in the control group (aRR = 1.50; 95% CI, 1.12–2.01; *P* = 0.007). However, this effect was not sustained at 12 months (aRR = 1.20; 95% CI, 0.85–1.69; *P* = 0.296).

Regarding the reduction in the number of health-risk behaviors, after adjustment for the same covariates, the intervention group demonstrated a 51% higher likelihood at 6 months (aRR = 1.51; 95% CI, 1.08–2.10; *P* = 0.016) and a 45% higher likelihood at 12 months (aRR = 1.45; 95% CI, 1.04–2.01; *P* = 0.027) compared with the control group.

In a sensitivity analysis, we adjusted for the stratification factors used in the randomization procedure (baseline diet, physical activity, alcohol use, and smoking status) in addition to age, sex, and education level. The results remained consistent with the primary findings (S2 Table). At 6 months, the intervention remained significantly associated with abstinence from ≥1 health-risk behavior (aRR 1.53, 95% CI 1.15–2.04, *p* = 0.004). For the secondary outcome of having reduced the total number of health-risk behaviors, the adjusted RR at 6 months was 1.51 (95% CI 1.09–2.10, *p* = 0.013), and at 12 months was 1.44 (95% CI 1.05–1.99, *p* = 0.026), remaining statistically significant.

In the post-hoc complete case analysis, the risk of abstinence from ≥1 health-risk behavior at 6 months was significantly higher in the intervention group compared with control group (RR, 1.30; 95% CI 1.01–1.67; *P* = 0.042), which was directionally consistent with but attenuated compared with the ITT estimate. At 12 months, the CCA estimate was similarly non-significant (adjusted RR 1.03, 95% CI 0.76–1.40; *P* = 0.83). Results of the CCA are presented in S3 Table.

In a sensitivity analysis using multiple imputation to account for missing outcome data, the intervention remained significantly associated with abstinence from ≥1 health-risk behavior at 6 months after adjustment for age, sex, education level, and baseline health-risk behaviors (aRR = 1.32, 95% CI 1.05–1.66, *p* = 0.018; S4 Table). The estimated effect size was attenuated compared with the primary intention-to-treat analysis but remained consistent in direction and statistical significance.

The observed pattern of outcomes, including higher abstinence and a greater reduction

in the number of health‑risk behaviors at 6 months, supports the underlying intervention logic that early, autonomy‑supported success facilitates subsequent behavior change. The reduction in effects following cessation of booster messaging suggests that continued reinforcement is important for sustaining change.

Table 4 summarizes changes in specific health-risk behaviors at 6- and 12-month follow-ups. At 6 months, the improvement in abstaining from physical inactivity was significantly greater in the intervention group than in the control group (66/208 [31.7%] versus 32/198 [16.2%]; RR, 1.96; 95% CI, 1.35–2.86; adjusted *p* = 0.002). This effect was not sustained at 12 months (44/208 [21.2%] versus 32/198 [16.2%]; RR, 1.31; 95% CI, 0.87–1.98; adjusted *p* = 0.80).

**Table 4:** Specific health-risk behavior changes at 6 and 12 months (*n* = 572).

Outcomes	No./Total No. (%)				
	Total	Control	Intervention	*p* ^a^	RR (95% CI)^c^
Changed to healthy diet					
6 months	42/515 (8.2)	22/256 (8.6)	20/259 (7.7)	1.00	0.90 (0.50–1.61)
12 months	28/515 (5.4)	15/256(5.9)	13/259 (5.0)	1.00	0.86 (0.42–1.77)
Changed to adequate physical activity					
6 months	98/406 (24.1)	32/198 (16.2)	66/208 (31.7)	**0.002**	1.96 (1.35–2.86)^**^
12 months	76/406 (18.7)	32/198(16.2)	44/208 (21.2)	0.80	1.31 (0.87–1.98)
Changed to no binge drinking					
6 months	6/62 (9.7)	4/39 (10.3)	2/23 (8.7)	1.00^b^	0.85 (0.17–4.33)
12 months	7/62 (11.3)	4/39 (10.3)	3/23 (13.0)	1.00^b^	1.27 (0.31–5.25)
Quit smoking					
6 months	9/89 (10.1)	3/45 (6.7)	6/44 (13.6)	0.85^b^	2.05 (0.54–7.73)
12 months	11/89 (12.4)	6/45 (13.3)	5/44 (11.4)	1.00	0.85 (0.28–2.61)

^a^Chi-squared test.

^b^Fisher exact test; ^c^ derived from a generalized estimating equation model with a binomial log link function using robust standard errors clustered by participant. All P values were adjusted for multiple comparisons using the Benjamini–Hochberg method (*m* = 8).

* *p* < 0.05; ** *p* < 0.01; *** *p* < 0.001.

Other health-risk behaviors, including unhealthy diet, binge drinking, and smoking, showed no statistically significant difference between the intervention and control groups at either the 6- or 12-month follow-ups (all *P* > 0.05).

## Discussion

This RCT demonstrated that a general health-promotion approach incorporating a brief AWARD-model intervention and mobile instant messaging support increased abstinence from health-risk behaviors among discharged ED patients. At 6 months, the primary endpoint, intervention participants were approximately 50% more likely than controls to achieve abstinence from at least one health-risk behavior and were also more likely to reduce the total number of health-risk behaviors. These findings support the concept that fostering a general intention to improve health may facilitate positive changes across multiple behavioral domains.

The interpretation of the primary findings should take into account the sensitivity of the estimated effect size to the handling of missing data. Although the complete-case analysis yielded a smaller effect estimate (aRR = 1.30) than the primary intention-to-treat analysis using the “missing as failure” assumption (aRR = 1.51), the direction of effect remained unchanged. To further evaluate the impact of missing data, we conducted multiple imputation with 40 imputations stratified by treatment group. The resulting estimate (aRR = 1.32, 95% CI 1.05–1.66, *p* = 0.018) was very similar to that obtained from the complete-case analysis and remained statistically significant. Together, these analyses suggest that the intervention effect is attenuated under alternative assumptions regarding missing data; however, the direction of the effect remains consistent across analytical approaches. These findings provide reassurance regarding the robustness of the results while highlighting the importance of considering missing-data assumptions when interpreting the magnitude of the treatment effect.

Approximately 71% of participants reported more than one health-risk behavior at baseline, confirming the substantial clustering of risk behaviors in this ED population and supporting the need for interventions that target multiple behavioral risks simultaneously [28]. Smoking and binge drinking showed the highest degree of clustering, consistent with prior evidence that individuals engaging in these behaviors are at elevated risk of developing NCDs [29,30]. Our findings extend previous work by Lam and colleagues [13,14] by demonstrating that a general health-promotion approach can be implemented effectively in the ED setting through a scalable mobile-health intervention.

Exploratory analyses suggested that participants with fewer baseline health‑risk behaviors may have achieved higher rates of behavioral change than those with multiple concurrent risk factors; however, the trial was not powered to formally test differences across risk‑factor strata, and these observations should be interpreted as hypothesis‑generating only.

This interpretation aligns with studies in Chinese populations by Lam and colleagues [13,14], who showed that a general intention to enhance health predicted abstinence from a targeted behavior and subsequent progression to abstain from additional behaviors. Our results extend this concept to the ED as a novel clinical setting and demonstrate its feasibility through a scalable, digitally augmented intervention.

Despite the significant benefit observed at 6 months, the intervention effect was not sustained at 12 months. Although abstinence rates remained numerically higher in the intervention group, the between-group difference was no longer statistically significant. Overall, 63.6% of participants who achieved abstinence from at least one health-risk behavior at 6 months relapsed by 12 months, whereas relapse was uncommon among those who reduced the total number of risk behaviors. The attenuation of effects likely reflects the cessation of booster messaging after the active intervention period and suggests that ongoing reinforcement may be necessary to maintain behavior change over time.

Importantly, the absence of a sustained intervention effect at 12 months suggests that the observed benefits may be time-limited. While the intervention was associated with beneficial effects during the active intervention period, additional reinforcement or maintenance strategies may be required to sustain these effects over the longer term. This pattern suggests that the intervention primarily produced short-term benefits, with limited evidence of sustained effects beyond the active intervention period.

At 12 months, the proportion of intervention participants abstaining from at least one behavior remained higher than in the controls, but the difference was no longer statistically significant. This attenuation is plausibly due to the cessation of booster messaging after six months, as sustained change, particularly across multiple behaviors, typically requires ongoing support and extended intervention periods, often up to one year. Future work should evaluate the effectiveness of prolonged booster delivery and extended follow up at strengthening sustainability. These findings should be interpreted with caution, as the 12-month results did not demonstrate a sustained intervention effect. The evidence for long-term efficacy therefore remains limited, and the observed benefits were primarily evident during the active intervention period.

Physical inactivity was the only individual behavior showing a significant intervention effect. This may reflect the relative ease and flexibility of increasing physical activity compared with changing diet, smoking, or alcohol consumption. In contrast, the lack of effect for dietary change may partly reflect a ceiling effect because 90% of participants reported unhealthy dietary habits at baseline, leaving limited room for measurable improvement. Smoking and binge drinking are inherently addictive behaviors and may require more intensive and tailored interventions. Moreover, subgroup sizes for smokers and binge drinkers were relatively small, limiting statistical power for behavior-specific analyses. As the trial was powered for the primary composite outcome rather than individual behaviors, these findings should be interpreted cautiously.

This RCT is the first, to our knowledge, to evaluate a theory-based general health-promotion approach combining AWARD-model counseling and mobile instant messaging support among ED discharge patients. The intervention is low-cost, scalable, and readily integrated into routine clinical workflows, highlighting the potential of the ED as a teachable moment for preventive health promotion.

This study has several limitations. First, outcomes were self-reported and therefore subject to reporting bias. Second, the study was conducted in a single public ED in Hong Kong, which may limit generalizability. Third, subgroup analyses for specific health-risk behaviors were constrained by relatively small sample sizes. The overall consent rate was modest and may have been influenced by prolonged waiting times in the ED.

Another limitation relates to outcome assessment. Although efforts were made to minimize detection bias by separating outcome assessment from intervention delivery and using standardized protocols, complete assessor blinding could not be guaranteed due to the behavioral nature of the intervention. Furthermore, while randomization achieved overall balance, some baseline imbalance in sex was observed; all analyses were adjusted accordingly to mitigate potential confounding.

A further limitation relates to the handling of missing outcome data. The baseline imputation approach (missing = failure) may have favored the intervention arm given higher attrition in the control group. However, sensitivity analyses using complete case analysis and multiple imputation yielded directionally consistent, although attenuated, effect estimates, supporting the overall interpretation of intervention benefit. Nevertheless, findings should be interpreted cautiously. Although follow-up completion rates were lower than the number of participants required per group in the original sample-size calculation, the primary intention-to-treat analysis retained all 572 randomized participants using the prespecified conservative assumption that missing outcomes represented no behavior change. Consequently, the trial remained adequately powered for the primary composite outcome, as evidenced by the statistically significant intervention effect at 6 months. However, attrition may have reduced power for complete-case, long-term, and behavior-specific subgroup analyses.

A further key limitation is the lack of a sustained treatment effect at 12 months. Although the intervention demonstrated beneficial effects at 6 months, these effects were not maintained at the 12-month follow-up. The findings therefore suggest that the intervention’s benefits may be time-limited, and that additional reinforcement or maintenance strategies may be required to sustain behavior change over the longer term. Future studies should evaluate extended intervention periods, maintenance approaches, or graduated withdrawal of support to determine whether more durable effects can be achieved.

Additional limitations include variation in intervention exposure among participants who subsequently targeted additional health-risk behaviors, the absence of process measures assessing behavioral intentions and perceived difficulty of behavior change, and the restriction of participation to individuals with smartphone access. Finally, resource constraints limited support to weekday daytime hours, suggesting that future integration of AI-assisted support tools may improve scalability and accessibility.

This innovative, low-cost, scalable intervention offers a pragmatic approach for integrating preventive care into routine ED practice with minimal additional resources. Importantly, the pattern of outcomes observed in this trial suggests that ongoing booster support plays a critical role in maintaining intervention effects. The attenuation of benefits after cessation of the 6‑month booster phase highlights the challenge of sustaining behavior change once structured support is withdrawn and underscores the need to explicitly address intervention durability in future designs.

A multicenter RCT with extended follow-up is warranted to confirm effectiveness, sustainability, and generalizability. Objective outcome measures, such as wearable-derived activity metrics and biochemical verification of smoking cessation, should complement self-reported data. Evaluations of cost-effectiveness and quality-of-life outcomes will also be important for informing clinical implementation.

Intervention effectiveness and sustainability may be further enhanced through brief behavior-specific videos delivered via mobile applications. Our previous work demonstrated that one-minute videos promoted smoking cessation among expectant fathers [31]. Such audiovisual content may improve engagement, reinforce key messages, and strengthen intentions to abstain from health-risk behaviors [32].

Tobacco use, harmful alcohol consumption, unhealthy diets, and physical inactivity are major contributors to NCDs. This trial showed that a digitally augmented general health-promotion strategy delivered after ED discharge can promote short-term abstinence from health-risk behaviors. However, the intervention effect was not sustained at 12 months, suggesting that additional reinforcement or maintenance strategies may be required to support long-term behavior change. Integration of this approach into routine ED workflows may provide a scalable strategy for NCD prevention, although further research is needed to optimize long-term effectiveness.

## Supporting information

S1 Checklist CONSORT 2025 Checklist. Hopewell S, Chan AW, Collins GS, Hróbjartsson A, Moher D, Schulz KF, et al. CONSORT 2025 Statement: updated guideline for reporting randomised trials. BMJ. 2025; 388:e081123. https://dx.doi.org/10.1136/bmj-2024-081123. 2025 Hopewell et al. This is an Open Access article distributed under the terms of the Creative Commons Attribution License (https://creativecommons.org/licenses/by/4.0/), which permits unrestricted use, distribution, and reproduction in any medium, provided the original work is properly cited.
(DOCX)

S1 Text Study protocol. (PDF)

S2 Text Intervention, theoretical framework, participant flow, and outcomes. (DOCX)

S3 Text Content of the weekly WhatsApp/WeChat digital messages (English translation). (PDF)

S1 Table Criteria for determining each health-risk behaviour and for defining successful abstinence from these behaviours. (DOCX)

S2 Table Sensitivity analysis adjusted for stratification factors. (DOCX)

S3 Table Sensitivity analysis using complete case analyses. (DOCX)

S4 Table Sensitivity analysis using multiple imputation for missing data. (DOCX)

## Abbreviations

AI: artificial Intelligence
CCA: complete case analysis
ED: emergency department
GEE: generalized estimating equation
ITT: intention-to-treat
NCDs: noncommunicable diseases
RCT: randomized controlled trial
RR: risk ratio
RRs: relative risks
