# Supplementary material for: Effect of a digitally augmented general health promotion intervention on abstinence from health-risk behaviors among emergency department discharge patients: A randomized controlled trial
Source: PLoS Med. 2026 Sep 8;23(9):e1004916. doi: 10.1371/journal.pmed.1004916 (PMC13568513; doi:10.1371/journal.pmed.1004916)
Supplement: Supplementary file 1 [file pmed.1004916.s002.pdf]

A. **Title**: A general-health-promotion-approach to promoting healthier lives among discharged emergency department attendees: A randomized clinical trial

B. Introduction

Hong Kong is facing a growing burden of non-communicable diseases (NCDs), including cardiovascular diseases, cancer, diabetes and chronic respiratory diseases, exacerbated by an aging population.[1] In 2016, 25,771 registered deaths (approximately 55% of all deaths) were attributed to NCDs.[2] In addition, NCDs caused 104,600 potential years of life lost before the age of 70.[1]

The WHO has identified four major behavioral risk factors that substantially contribute to NCDs and can increase the risk of death; these are tobacco use, excessive alcohol use, an unhealthy diet and physical inactivity.[3] Most premature deaths from NCDs could have been prevented through lifestyle modification.[4] Therefore, assisting people to engage in healthy lifestyle practices, such as quitting smoking, avoiding excessive alcohol use, maintaining a balanced diet and engaging in regular physical activity, can help prevent NCDs and improve the overall health of the population. However, many people lack motivation or find it difficult to change their health-risk behaviors, especially when there is little advice and support from healthcare professionals.[5-8]

The receipt of medical attention at an emergency department (ED) by a person in physical discomfort can be an excellent 'teachable moment' because it provides an invaluable opportunity to initiate healthy lifestyle practices. People who consult a doctor in an emergency are more liable to change their risk behaviors to improve their health. According to the Hospital Authority Annual Report,[9] there were 1.8 million EDs visits in Hong Kong between 2021 and 2022. More than half of all emergency department visits were triaged as semi-urgent (level 4) or non-urgent (level 5), and most were discharged home after seeking medical attention. A study investigating the prevalence of elevated blood pressure among ED patients in Hong Kong found that among all patients admitted and discharged on the same day, 86.6% had elevated blood pressure > 160/100 before discharge.[10] Furthermore, more than 75% of these patients had no referral or follow-up.[10] Evidence suggests that health-risk behaviors including tobacco use, excessive alcohol use, an unhealthy diet and physical inactivity are risk factors for hypertension.[1,11] According to our pilot study conducted in the ED of United Christian Hospital, more than 70% of attendees triaged as semi-urgent or non-urgent had at least one health-risk behavior. Therefore, it is crucial to provide health advice to help emergency department attendees with health-risk behaviors lead healthier lives prior to discharge. However, the clinical environment in Hong Kong's EDs is extremely busy, and nurses working there often claimed that they do not have even a few minutes to provide detailed health advice to discharged patients.

Previous studies have shown that individuals with a general intention to improve their health are more likely to engage in desirable health-related lifestyle practices, and once engaged, will progress to other healthy lifestyle practices[12,13] Based on this concept and results, we conduct a pilot study with the funding from the Health and Medical Research Fund (#16172831) to evaluate a general health promotion approach delivered via instant messaging (WhatsApp or WeChat) to help smokers who had medical follow-up in a special out-patient clinic quit smoking.[14] The pilot study found that more than 90% of the participants owned a smartphone and could use an instant messaging application.[14] The participants were motivated to first choose and engage in any desirable health-related lifestyle, such as exercising regularly or maintaining a healthy diet, and then eventually to quit smoking. The results of this pilot study demonstrated the feasibility and potential benefits of a general health-promotion approach to motivate smokers who have no initial intention of quitting to eventually take action to quit.[14] However, it is uncertain whether such approach can help discharged emergency department attendees with health-risk behaviors lead healthier lives. Large-scale RCTs are warranted to provide strong evidence of the efficacy of this approach.

C. <u>Aim and Hypotheses to be Tested:</u>

<u>Aim</u>

To examine the effectiveness of a general-health-promotion-approach to help ED attendees change their health-risk behaviors and lead healthier lives.

<u>Hypotheses to be tested</u>

We hypothesize that compared with those in the control group, the participants in the intervention group will experience significantly greater success in changing their health-risk behaviors at 6 months follow-up.

[World count: 692]

D. Plan of Investigation:

(i) <u>Subjects</u>

Chinese patients who attend the ED because of physical discomfort and who fulfil the following criteria will be invited to participate. Inclusion criteria include (1) age 18–65 years, (2) triage as semi-urgent (level 4) or non-urgent (level 5) and will be discharged home the same day after seeking medical attention, and (3) the presence of at least one health risk behavior (tobacco use, harmful use of alcohol, unhealthy diet, and physical inactivity), and (4) able to communicate in Chinese. Exclusion criteria include (1) poor cognitive state or mental illness and (2) diagnosed with NCDs and have regular follow-up

in outpatient clinics, (3) participation in another related study. Individuals who did not have a smartphone or who did not use WhatsApp/WeChat (unlikely in Hong Kong) were provided brief advice and a Practical Resource Hub for Healthy Life leaflet on healthy lifestyles but were otherwise excluded from the study.

(ii) Methods

Setting:

The setting is the ED of the United Christen Hospital (UCH), one of the major acute care hospitals in Kowloon East cluster in Hong Kong.

Conceptual and theoretical framework:

The proposed intervention is guided by the foot-in-the-door technique, and self-determination theory.

Foot-in-the-door technique

The foot-in-the-door technique, which was introduced by Freeman and Fraser,[21] emphasizes the notion that individuals who initially comply with a small, easy request are more likely to later comply with a larger request.[15] Compliance with the first request or target increases the individual's confidence and alters their self-perceived capability and willingness regarding further requests or targets. This technique can facilitate the recruitment process and enhance compliance.

Self-determination theory

According to self-determination theory,[16] behavioral regulation is more autonomous when it is internalized, as opposed to being regulated by external factors (e.g., orders from family members, friends, or healthcare professionals). Compared with external regulation, autonomous regulation is associated with increased self-efficacy, greater behavioral persistence, longer-term behavioral changes and more positive health behaviour.[17] Autonomy is another influential determinant of behavior that is emphasized by freedom of choice.[16] There is some evidence that supports a positive association between autonomy and competence,[16,17] with people who have greater autonomy demonstrating greater competence in achieving behavioral change compared with those with less autonomy. Evidence suggests that patients who have the opportunity to decide on their own treatment may be more eager to comply with instructions.[18] The participants in this proposed study will be allowed to select their priorities in engaging in desirable health-related lifestyle practices after discussion with the research assistant. It is anticipated that the participants will show a greater willingness to adhere to their desired health-related lifestyle practice as a result of this increase in autonomy. Moreover, there is some evidence for a positive association between autonomy and self-efficacy;[19] that is, people who have greater autonomy demonstrate greater self-efficacy for achieving behavioral change. As a result, increased autonomy will facilitate a gradual change in risky behaviors.

Our intervention, therefore, aims to first change the participants' attitudes and their subjective norms through risk communication. By using the foot-in-the-door technique and self-determination theory, it will then enhance the participants' self-efficacy in changing their health-risk behaviors.

Intervention

At the ED

All potential participants will be approached by emergency nurses before being discharged home from the ED. The emergency nurse will provide potential participants with a leaflet detailing the nature and purpose of the study. Potential participants can express their willingness to participate in the study by signing an informed consent form. The emergency nurse will then refer the participants to the research assistant. Participants will be informed that they will receive a telephone call from a research assistant within 3 days to evaluate their potential health-risk behaviors and provide them with appropriate health advice to lead healthier lives. The participants will also be given a Practical Resource Hub for Healthy Life leaflet containing information on various applications, including (i) 'Move Your Body', (ii) 'Eat Healthy', (iii) 'Live Alcohol Free', and (iv) 'Stay Away from Tobacco', which were developed by the Hong Kong Department of Health.

(a) *Intervention group*

Brief intervention via telephone (within 3 days after visiting the ED)

The participants will receive a brief intervention using the Ask, Warn, Advise, Refer and Do-it-again (AWARD) model, which was originally developed for primary-care tobacco-cessation interventions. The brief intervention includes the following steps: (1) ask about and assess health-risk behaviors; (2) warn about the high morbidity and mortality risks associated with health-risk behaviors; (3) advise to adopt healthy lifestyle practices to benefit the health of the participant; (4) refer to hotline services on request, such as smoking cessation and alcohol treatment services; and (5) do it again. For the warning message, the research assistant will provide the following standardized message: 'The WHO has identified four major behavioral risk factors, namely, tobacco use, harmful alcohol use, an unhealthy diet and physical inactivity, that contribute substantially to NCDs, which result in high rates of morbidity and mortality.' For the advice step, the research assistant will ask about the priority the participants place on engagement in desirable health-related lifestyle practices identified in the completed behavioral risk factor survey. The participants will also be asked to choose the goal that they consider easiest to achieve, such as quitting or reducing smoking, consuming more vegetables or less fatty foods or sugary drinks, performing more exercise or reducing alcohol consumption. The participants will be encouraged to quit health-risk behaviors (or adopt a healthy lifestyle) sequentially, but they will also be able to choose to quit them simultaneously if they are confident in doing so. Each participant will then receive a brief (approximately 5 minutes), individual intervention with health advice about the

selected health-related lifestyle practice. The whole intervention will last approximately 10 minutes, but slightly longer if necessary. Such a brief intervention will be more feasible for routine use in clinical practice by healthcare professionals after minimal training. Previous clinical trials of smoking cessation have provided strong evidence regarding the effectiveness of brief interventions using the AWARD model.[6,7,18]

At the end of the telephone call, the participants will be informed that throughout the study period, the research assistant will assist in achieving the participant's health-related goals by sending WhatsApp/WeChat messages or via telephone calls.

Follow-up booster intervention (up to 6 months)

For the first 6 months of the study period, the research assistant will deliver WhatsApp/WeChat messages about once per week. If a participant does not own a smartphone or is unable to receive WhatsApp/WeChat messages (unlikely in Hong Kong), the research assistant will provide brief advice and a Practical Hub for Healthy Life leaflet on healthy lifestyles but will be otherwise excluded from the study. Instant messaging via mobile applications has been found to be effective in enhancing treatment compliance.[20]

Follow-up assessment of behavioral changes at 3, 6, and 12 months

The participants will be assessed to determine their success in changing their targeted health-related lifestyle practice via a phone call (please see Appendix 1 for the criteria for successfully quitting health-risk behaviors or engaging in a desirable health-related lifestyle) at 3, 6, and 12 months.

(b) Control group

The control group participants will receive a brief telephone intervention based on the AWARD model and delivered by the trained research assistant, similar to that delivered to the intervention group. However, the research assistant will simply advise the participants to change their health-risk behaviors and/or adopt a healthy lifestyle practice. The participants will also receive follow-up for outcome assessments with the same schedule as those in the intervention group.

(iii) Study design

A RCT with a two-group pre-test and repeated post-test between- subjects design will be conducted.

Subject recruitment

We will obtain ethical approval from the institutional review board of the Kowloon East cluster. Potential participants will be assessed for eligibility, and the purpose, design, procedures, and potential benefits and risks of the study will be explained to them. Informed written consent will then be sought. All subjects will be assured that their

participation will be voluntary, with no prejudice attached to refusal, and that the information they provide will be kept confidential. The researcher is responsible for its safekeeping and the only one who would have access to the raw data or study record during or after the study. After the period of storage, all raw data and records will be destroyed. The proposed study will adhere to the Declaration of Helsinki − Ethical Principles for Medical Research Involving Human Subjects.[26]

Randomization and allocation concealment

To avoid the potential risk of treatment contamination within the ED, randomization was not performed there. Instead, the research assistant inputted the baseline data collected at the ED directly into the web-based trial entry form linked to a computerized database, and randomization was performed at the principal investigator's institution by an independent statistician who had no other involvement in the study. Stratified block randomization with 1:1 allocation was conducted using varying block sizes of 4–8 to achieve an appropriate balance of participant numbers between the two groups and to optimize allocation concealment. Randomization was stratified by health-risk behaviors (tobacco use, harmful use of alcohol, unhealthy diet, and physical inactivity) to yield a balanced allocation of participants with different lifestyle risk profiles between the two groups.

(iv) Data processing and analysis

Instruments:

A behavioral risk-factor survey will be used to collect the eligible participants' demographic and clinical data and information about their health-risk behaviors at baseline and at 3, 6, and 12 months. This questionnaire is adapted from a questionnaire used by the Hong Kong Department of Health and was used to investigate multiple health-risk behaviors among Chinese adults in Hong Kong in 2021.[21]

All the baseline data will be collected by a research assistant during the daytime. In addition, details of the study, consent form and information sheet, and the questionnaire will be installed on tablet computers and hung on the wall in a prominent place of the ED. In the absence of the research assistant, anyone who wants to participate in the study can do it themselves through the tablet computer. The tablet is user friendly. Furthermore, such systems have built-in functions to protect against errors and allow the collected data to be easily processed for progress monitoring and analysis. A research assistant will then contact subjects by phone and provide them with health-related lifestyle advice and follow-up.

Outcome measures:

(1) Efficacy the proposed intervention

A behavioral health-risk factor survey will be used to collect information regarding health-risk behaviors. The demographic data included age, gender, socioeconomic status, and presence of NCDs. We will assess any changes in four major health-risk behaviors— (1) smoking, (2) alcohol consumption, (3) physical inactivity and (4) unhealthy diet—at 3-, 6-, and 12-months. The primary outcome will be abstinence from at least 1 health-risk behavior at 6 months. The criteria are presented in Appendix 1. The secondary outcomes will be abstinence from at least 1 health-risk behavior at 12 months and reduced number of total health-risk behaviors at 6 and 12 months.

Sample size

G*Power is used to estimate the sample size based on the results of our pilot study.[14] The pilot study showed an approximately 10% between-group difference in reports of successfully abstinence from at least one health-risk behavior between groups at 12 months. To detect a significant difference in the proportion of participants quitting health-risk behaviors or adopting healthy lifestyle practices between the intervention and control groups, with a power of 80% and a significance level of 5% (2-tailed), at least 199 participants will be needed per group. To account for a potential attrition rate of 30% at the 12-month follow-up assessment, at least 568 participants will be recruited.

According to the Hospital Authority Annual Report,[9] there were 140,273 visits in ED of UCH between 2021 and 2022. Furthermore, according to our previous pilot study conducted in ED of UCH, we are confident that we will be able to recruit the required number of participants (N =573) within 15 months.

Data Analysis

The baseline characteristics of the two groups will first be compared using the chi-square test for categorical variables and analysis of variance for continuous variables. An intention-to-treat analysis will be used by imputing all non-responses at follow-up by baseline values (i.e. assuming failure or no change after the intervention), to yield more conservative effect size estimates.

SPSS for Windows (SPSS version 26.0; IBM Corp., Armonk, NY, USA) will be used for the quantitative data analysis. Descriptive statistics will be used to calculate the mean, standard deviation, and frequency of the demographic and health-risk behavior data. The primary analysis including the main effect, i.e., behavioral changes at 6 months in the intervention group versus those in the control group, will be performed using a chi-square test or Fisher's exact test. The secondary analyses will (1) assess the main effect adjusted for baseline differences, (2) assess the secondary outcome (abstinence from at least 1 risky behavior at 12 month and having reduced total number of health-risk behaviors at

6 and 12 months), and (3) construct a generalized estimating equation model (GEE). The GEE model will be used to calculate the adjusted odds ratios for successful abstinence of at least 1 health-risk behavior and having reduced the total number of health-risk lifestyles, after adjusting for the baseline demographic and clinical characteristics that show significant differences, and the within-subjects effect of the repeated-measure outcomes (6 and 12 months). GEE models were used to assess the differences in proportions of participants who abstained from any specific health-risk behavior at 6 and 12 months.

[World count: 2323]

E. <u>Potential contributions to clinical practices</u>

According to the WHO, tobacco use, excessive alcohol consumption, physical inactivity, and unhealthy diet increase the risk of NCD-related mortality. Moreover, the high mortality and morbidity rates associated with NCDs greatly impact the health of the affected individuals, and burden their families, the healthcare system, the economy and society. Therefore, a new approach is needed to motivate more individuals to quit their health-risk behaviors and lead healthier lives.

It is anticipated that this study will demonstrate the effectiveness of a proactive approach using a brief self-determination theory-based intervention to help people attending the ED change their health-risk behaviors and lead healthier lives. Our proposed novel approach and intervention have excellent potential to be effective and inexpensive, making important contributions to evidence-based medicine for reducing risk and promoting healthy behaviors. Our results will inform future researchers and policymakers regarding the prevention and control of NCDs and will thus have important implications for clinical practice and health enhancement.

The results of our proposed project will be published in renowned international peer-reviewed journals and will be disseminated to the public through local and international conferences, such that healthcare professionals can replicate the intervention within their clinical practice to promote healthy lifestyles. Moreover, the implementation of our intervention using a primary healthcare approach will improve the early detection and timely treatment of NCDs. As the intervention will be administered to the patients during the early stages of their NCD, it will reduce the need for more expensive treatments, resulting in economic benefits.

The findings of the proposed project will facilitate the development of new evidence-based proactive interventions with real-world effectiveness for individuals with health-risk behaviors. Moreover, the findings will promote the development of clinical practice guidelines for healthcare professionals to deliver the intervention in the clinical and community settings.

[World count: 295]

References:

1. Hong Kong. Food Health Bureau, issuing body. *Towards 2025: Strategy and Action Plan to Prevent and Control Non-Communicable Diseases in Hong Kong*. [Department of Health], 2018.
2. 2016 Statistics of Inpatient Discharges and Deaths. Hong Kong SAR: Hospital Authority, Department of Health and Census and Statistics Department.
3. World Health Organization, Global Action Plan for the Prevention and Control of Noncommunicable Diseases 2013-2020. 2013. Available online: http://apps.who.int/iris/bitstream/10665/94384/1/9789241506236_eng.pdf?ua=1. (accessed on 1 May 2023)
4. GBD 2015 Risk Factors Collaborators. Global, regional, and national comparative risk assessment of 79 behavioural, environmental and occupational, and metabolic risks or clusters of risks, 1990–2015: a systematic analysis for the Global Burden of Disease Study 2015. Lancet, 2016; 388(10053):1659-1724.
5. Li HCW, Ho KY, Xia VW, Wang MP, Lam KK, Chan SS, Lam TH. Helping hospitalized smokers in Hong Kong quit smoking by understanding their risk perception, behaviour, and attitudes related to smoking. J Adv Nurs. 2019;75(10):2167-77.
6. Li HCW, Wang MP, Ho KY, Lam KWK, Cheung YTD, Cheung TY, Lam TH, Chan SSC. Helping cancer patients quit smoking using brief advice based on risk communication: A randomized controlled trial. Sci Rep. 2018, 8:2712.
7. Li HCW, Wang MP, LAM TH, Cheung TY, Cheung YTD, Suen YN, Ho KY, Tan KCB, Chan SSC. Brief intervention to promote smoking cessation and improve glycemic control in smokers with type 2 diabetes: a randomized controlled trial. Sci Rep. 2017; 7: 45902.
8. Chan SS, Leung DY, Wong DC, Lau CP, Wong VT, Lam TH. A randomized controlled trial of stage-matched intervention for smoking cessation in cardiac out-patients. Addiction. 2012;107:829–37.
9. Hospital Authority. "Hospital Authority annual report 2021–2022." (2022).
10. Graham CA, Chan SS, Ahmad I, Rainer TH. 405 Elevated Blood Pressure in Emergency Department Patients in Hong Kong: Prevalence, Characteristics and Disposition. Annals of Emergency Medicine, 2012; 60(4): S142-S143.
11. Tsoi LCH, Tung CCH, Wong ELY. "Nurse-led hypertension referral system in an emergency department for asymptomatic elevated blood pressure." Hong Kong Med J 2012: 201-6.
12. Lam TH, Chan B, Ho SY, Cha WM. Stage of change for general health promotion

action and health-related lifestyle practices in Chinese adults. PrevMed, 2004; 38:302–8.

13. Lam TH, Chan BH, Chan SK, Leung GM, Ho SY, Chan WM, et al. Prospective study of stage of change for general health promotion action and health-related lifestyle practices among Chinese adults. Soc Sci Med, 2006; 63:1846–56.
14. Li HCW, Ho LLK, Cheung AT, Wang MP, Cheung YT, Xia W, Lam TH. A general health promotion approach to helping smokers with non-communicable diseases quit smoking: A pilot randomized controlled trial. Front. Public Health, 2022;10.
15. Freedman JL, Fraser SC. Compliance without pressure: the foot-in-the-door technique. J Pers Soc Psychol.1966; 4:195.
16. Deci E, Ryan R. *Handbook of self-determination research*. Rochester, NY: University of Rochester Press, 2002.
17. Bandura A. *Self-efficacy: The exercise of control*. New York: Freeman, 1997.
18. Li HCW, Ho KY, Wang MP, Cheung DYT, Lam KWK, Xia W, Cheung KY, Wong CKH, Chan SSC, Lam TH. Effectiveness of a brief self-determination theory-based smoking cessation intervention for smokers at emergency departments in Hong Kong: a randomised controlled trial. *JAMA Internal Medicine.* 2019; 180(2), 206–214.
19. Williams GC, McGregor HA, Zeldman A, *et al*. Testing a self-determination theory process model for promoting glycemic control through diabetes self-management. *Health Psychology* 2004; **23**: 58-66.
20. Krishna R. The Impact of Health Information Technology on the Doctor-Patient Relationship in Child and Adolescent Psychiatry. Child Adol Psych Cl. 2017; 26: 67-75.
21. Li HCW, Ho LLK, Chung OKJ, Cheung AT, Xia W, Song P. A Descriptive Study on Multiple Health-Risk Behaviors among Chinese Adults in Hong Kong. Int J Environ Res Public Health. 2022; 19(18): 11393.

Appendix 1

Criterion for determination of each health-risk behaviour

**Behavioural risk factors**	**Criterion of health-risk behaviour**
Tobacco use[1]	Smoked at least 1 cigarette a day over the past 30 days
Binge drinking[2]	Reporting ≥1 binge drinking episodes (consumption of five or more alcoholic beverages on one occasion) in the past month.
Unhealthy diet[3]	Have consumed less than five servings of fruit and vegetables per day or have had a daily intake of less than 400 grams of fruit and vegetables
Physical inactivity[4]	(1) Performed less than 150 minutes of moderate-intensity aerobic physical activity throughout the week, or (2) performed less than 75 minutes of vigorous-intensity aerobic physical activity throughout the week

The criteria for successfully changing health-risk behaviours or engaging in a desirable health-related lifestyle practice

**Behavioural risk factors**	**Successful criteria of quitting health-risk behaviours or engaging in healthy lifestyle practice**
Tobacco use[1]	Self-reported 7-day point prevalence of smoking abstinence
Binge drinking[2]	Self-report no binge drinking episode (consumption of five or more alcoholic beverages on one occasion) in the past month.
Unhealthy diet[3]	Have consumed at least five servings of fruit and vegetables per day or have had a daily intake of at least 400 grams of fruit and vegetables
Physical inactivity[4]	Performed at least 150 minutes of moderate-intensity aerobic physical activity throughout the week, or performed at least 75 minutes of vigorous-intensity aerobic physical activity throughout the week

References

1. Wang MP, Suen YN, Li WH, et al. Intervention with brief cessation advice plus active referral for proactively recruited community smokers: a pragmatic cluster randomized clinical trial. JAMA Intern Med. 2017;177(12):1790-1797.
2. Lam TH, Chan B, Ho SY, Chan WM. Stage of change for general health promotion action and health-related lifestyle practices in Chinese adults. Preventive Medicine. 2004; 1;38(3):302-8.

3. Fruit, vegetables and NCD disease prevention. Geneva: World Health Organization; 2003. (http://www.who.int/dietphysicalactivity/media/en/gsfs_fv.pdf).

4. Global Recommendations on Physical Activity for Health", World Health Organization; 2010. (http://whqlibdoc.who.int/publications/2010/9789241599979_eng.pdf)
