# Supplementary material for: Effect of a digitally augmented general health promotion intervention on abstinence from health-risk behaviors among emergency department discharge patients: A randomized controlled trial
Source: PLoS Med. 2026 Sep 8;23(9):e1004916. doi: 10.1371/journal.pmed.1004916 (PMC13568513; doi:10.1371/journal.pmed.1004916)
Supplement: Supplementary file 2 [file pmed.1004916.s004.pdf]

S3 Text Content of the weekly WhatsApp/WeChat digital messages (English translation).

No.	Cut down alcohol drinking	Quit smoking	Do enough exercise	Eat enough fruits and vegetables
1	Quitting alcohol is never easy, but if you look at it from a different perspective, what you're really doing is changing a habit.<br>If your health goal is to reduce alcohol consumption, there is an Alcohol Cessation Counselling Pilot Programme available. Feel free to find out more if you think it may be helpful.<br><br>This week, let's take a closer look at our goal and start with the very first step!<br><br>Breaking the habit: Try a different mindset. Ask yourself, "Let me run an experiment and see what changes I notice in my body if I don't drink for a week." Keep a record of your emotions and experiences, then review	"Can Fruits and Vegetables Help You Quit Smoking?"<br>Compared with non-smokers, smokers tend to consume more calories, total fat, saturated fat, cholesterol, and alcohol, while consuming fewer antioxidant vitamins and less dietary fiber. Adults should consume 3 to 5 servings of vegetables daily (approximately 3 to 5 fist-sized portions of cooked vegetables). Studies show that people who consume sufficient fruits and vegetables while quitting smoking are up to three times more likely to succeed.<br>Vitamin A Helps maintain the integrity of the oral mucosa and reduces damage caused by smoking.<br>Vitamin B Supports the nervous system, boosts energy levels, relieves low mood and fatigue, and may even improve sleep quality. Foods rich in B vitamins include whole grains, eggs, meat, and dairy products. Including a palm-sized portion of beans, fish, eggs, or meat in each meal can provide 1.5 to 2 servings of high-quality protein.<br>Vitamin C Smoking a single cigarette can deplete approximately 25 mg of	How Can You Build an Exercise Habit?<br><br>Enjoying exercise and avoiding psychological or physical burden are key factors in maintaining a long-term exercise routine. Therefore, it is recommended to start with light-intensity activities and gradually increase the intensity over time. This helps build a sense of achievement while preserving the enjoyment of exercise.<br><br>You can also increase your daily activity levels through simple lifestyle changes, such as:<br><br>Getting off public transportation one stop earlier and walking the remaining distance.<br>Taking the stairs instead of escalators or elevators.<br><br>Would you like to know what aerobic exercises you can do at home?	A reminder of the healthy lifestyle goal we set together: eat 5 servings of fruits and vegetables (80g each) every day!<br><br>How can you get enough nutrients through a healthy diet to support your immune system?<br><br>Consuming sufficient dietary fiber helps promote gut health. Foods rich in fiber include vegetables, fruits, and whole grains such as quinoa, oats, and brown rice.<br><br>Dietary fiber can also improve the balance of gut microbiota and help manage blood glucose, cholesterol, and blood pressure levels. A healthy gut microbiome supports the immune system by regulating immune responses and improving immune function.<br><br>Adults are recommended to consume approximately 25 grams of dietary fiber daily. Would you like to know how much food that actually translates to?

	them after a week and identify the situations or triggers that make you want to drink.<br><br>Emotional reliance: When facing anxiety or stress, alcohol can become the only coping mechanism. Try developing new interests or revisiting old hobbies, and engage in activities that make you feel better, such as going to the gym or enjoying your favorite buffet meal.<br><br>Social pressure: Turning down invitations from friends may feel awkward. The things that are hardest to change often require the most effort. Don't force yourself. Instead, try reducing the number of drinking-related social gatherings you attend and prepare polite reasons in advance for declining drinks.	vitamin C in the body. Fruits are an excellent source of vitamin C. Aim to consume at least two servings of fruit each day to help keep your mouth fresh, strengthen immunity, and reduce smoking cravings.			

2	Quitting alcohol takes time and persistence, but success can be supported by following the "EARS" principle.<br><br>With the upcoming festive season, company gatherings, and social events with friends becoming more frequent, why not try setting a specific goal?<br><br>For example, you could commit to a "Dry Week" from the week before Christmas through New Year's Day. Alternatively, use the money you save from not drinking to buy gifts for your friends and family.<br><br>You can still have plenty of fun by choosing non-alcoholic beverages or mocktails instead!	How Healthy Eating Can Help Reduce Oral Cravings<br>Many people experience strong oral cravings during the quitting process. Choosing healthy food alternatives can help you resist temptation and increase your chances of successfully quitting smoking.<br>Vegetables and Fruits Rich in fiber and water, they help you feel fuller and reduce cigarette cravings. Aim for five servings of fruits and vegetables daily.<br>Herbal Tea Avoid caffeinated or alcoholic beverages. Instead, try caffeine-free herbal teas such as peppermint or chamomile tea, which can help you relax, reduce anxiety, and replace the habit of smoking.<br>Dark Chocolate Enjoying moderate amounts of dark chocolate may improve mood and reduce cravings. Choose varieties with at least 70% cocoa content for greater health benefits.<br>Healthy Snacks Nuts and seeds contain healthy fats and protein, helping stabilize blood sugar levels and reduce hunger-related cravings.<br>Stay Hydrated Drinking plenty of water helps maintain hydration and reduces oral cravings, making it easier to resist the urge to smoke.	8 Household Chores Ranked by Calories Burned<br>Household chores can increase physical activity and help burn calories. Research has found that completing two hours of household chores can burn approximately 600 calories, even more than the 374 calories burned from running 5 kilometers.<br>8) Kitchen Cleaning (69 calories per 30 minutes)<br>7) Dishwashing (69 calories per 30 minutes)<br>Dishwashing is not just an arm workout. You can also strengthen your glutes and thighs by performing leg lifts while washing dishes. Lift one leg backward with a slight bend in the knee, then alternate sides. This helps tone the lower body.<br>6) Scrubbing the Bathtub (83 calories per 30 minutes)<br>Removing dirt from a bathtub requires effort and can also work your biceps.<br>5) Sweeping and Mopping (100-145 calories per 30 minutes)<br>Using a vacuum cleaner may be easier, but sweeping and mopping for 30 minutes can burn up to 145 calories, comparable to spending 15 minutes on a treadmill.<br>4) Hand Washing Laundry (116 calories per 30 minutes)	Easy Ways to Meet Your Daily Fiber Needs<br><br>Sources of soluble fiber include beans, oats, and certain fruits such as apples and pears. Insoluble fiber is found in whole-grain foods, wholemeal bread, and some vegetables.<br><br>To consume enough fiber throughout the day, try the following:<br><br>Breakfast or Afternoon Snack<br>One bowl of oatmeal<br>(10.2g of dietary fiber)<br>Lunch or Dinner<br>One bowl of brown rice<br>(4.4g of dietary fiber)<br>One bowl of broccoli<br>(6.4g of dietary fiber)<br>Fruit Options<br>One apple<br>(4.4g of dietary fiber)<br>One banana<br>(3.1g of dietary fiber)<br><br>Eating more fruits and vegetables supports digestion and gut health. Remember your goal of 5 servings of fruits and vegetables every day and take action toward this year's health goals!

			Washing, lifting, and hanging clothes can be surprisingly demanding. An hour of laundry activities may burn around 78 calories, equivalent to performing about 100 sit-ups.<br>3) Vacuuming (129 calories per 30 minutes)<br>Cleaning difficult areas such as the tops of cabinets and underneath furniture requires moving around and engaging multiple muscle groups. Rearranging furniture also increases the workout effect.<br>2) Cleaning Windows (172 calories per 30 minutes)<br>Window cleaning is excellent for toning the arms. Repetitive circular movements engage the back and abdominal muscles as well.<br>1) Taking Out the Trash Using the Stairs (249 calories per 30 minutes)<br>Try walking farther when taking out the trash and use the stairs instead of the elevator.	

3	Do you remember the healthy lifestyle goal we set together to cut down on drinking?<br>If you choose to drink alcohol, remember to stay hydrated by drinking 6 to 8 glasses of water a day.<br>An even better habit is to alternate between alcoholic and non-alcoholic drinks to gradually reduce your alcohol intake.	We understand that one of your health goals is to quit smoking as early as possible.<br>Did you know that quitting smoking not only reduces your risk of stroke and coronary heart disease but also saves money? If you spend around HK$60 on a pack of cigarettes every day, quitting could save you more than HK$20,000 per year!<br>Increasing your daily physical activity is recommended. Exercise releases endorphins, which help reduce stress and anxiety while shifting your attention away from cigarettes.<br>Aerobic exercise is especially beneficial for cardiovascular health.<br>Have you heard of diaphragmatic breathing?<br>By focusing on slow, deep breaths, you can relax your muscles, increase oxygen levels in your body, improve blood circulation, and reduce feelings of tension and anxiety. Taking care of your lungs means no longer letting nicotine control your health.	Aerobic Exercises Beginners Can Do at Home<br>Jumping Jacks<br><br>A simple exercise requiring no special skills. Jump while spreading your arms and legs outward, then return to the starting position.<br><br>High-Knee Running in Place<br><br>Keep your head slightly raised, neck relaxed, chest up, and core engaged. Bend your elbows at 90 degrees and swing your arms naturally while alternately lifting your knees.<br><br>Chest Stretch<br><br>Stand upright and extend your arms straight in front of you with thumbs pointing upward. Keeping your arms straight, move them forward and backward while maintaining them parallel to the floor.	Healthy dietary habits can significantly reduce the risk of stroke.<br>Fruits and vegetables are rich in antioxidants, fiber, and vitamins that help lower blood pressure and improve cardiovascular health. Dark leafy greens and berries are especially beneficial.<br>Consuming five servings of fruits and vegetables daily can help improve blood pressure control, support heart health, and boost overall energy levels.<br>Benefits of Unsaturated Fatty Acids<br>Monounsaturated Fatty Acids<br>Found mainly in:<br>Olive oil, Avocados, Nuts<br>These fats may:<br>Lower LDL ("bad") cholesterol<br>Increase HDL ("good") cholesterol<br>Reduce the risk of heart disease and stroke<br>Provide anti-inflammatory benefits<br>Polyunsaturated Fatty Acids<br>Especially Omega-3 and Omega-6 fats, found in:<br>Oily fish, Flaxseeds, Walnuts<br>These fats may:<br>Reduce inflammation<br>Support brain function<br>Improve mental well-being<br>Help reduce symptoms of depression<br>Eat deep-sea fish (such as salmon, mackerel, and sardines) at least twice a week.<br>Have a small handful of nuts and seeds

					daily.<br>Reduce saturated and trans fats by limiting red meat, full-fat dairy products, and processed foods.

4	Remember the healthy lifestyle goal we set together to cut down on alcohol drinking?<br>Try healthier alternatives such as sparkling water or alcohol-free cocktails to satisfy your cravings while reducing your reliance on alcohol. Every healthier choice brings you one step closer to your goal!	Don't Forget Your Goal to Quit Smoking!<br>Even a small step can bring about significant change!<br>We understand that different emotions can sometimes tempt you to take another puff. However, there are many simple strategies you can use to stay on track and keep moving toward your goal.	How to Effectively Set Exercise Goals<br><br>Starting an exercise journey can be both challenging and exciting. Whether your motivation is to improve health, build fitness, or boost your mood, taking the first step is something to be proud of.<br><br>Imagine jogging in the morning sunshine, breathing fresh air, or relaxing on a yoga mat and letting go of stress. Every drop of sweat is an investment in yourself, helping you become stronger and more confident.<br><br>Set Personalized Goals<br><br>Identify goals that are meaningful to you, whether it's weight loss, muscle gain, or improving cardiovascular fitness. Meaningful goals create stronger motivation.<br><br>Explore Different Activities<br><br>Try different forms of exercise such as Pilates, swimming, or dancing. When you find an activity you genuinely enjoy, exercise becomes something you look forward to.	A reminder of the healthy lifestyle goal we set together: 5 servings of fruits and vegetables every day!<br><br>Eating a variety of fruits provides different vitamins and minerals, which are essential for good health.<br><br>One fruit serving is approximately equivalent to:<br><br>Two small fruits (e.g., plums or kiwifruit)<br>One medium fruit (e.g., orange or apple)<br>Half a large fruit (e.g., banana, grapefruit, star fruit)<br>Half a bowl of chopped fruit (e.g., watermelon, cantaloupe, honeydew melon)<br>Half a bowl of small fruits (e.g., grapes, lychees, cherries, strawberries)<br>One tablespoon of unsweetened dried fruit (e.g., raisins or prunes)<br>Three-quarters of a cup of unsweetened pure fruit juice (e.g., fresh orange juice with pulp)<br><br>(1 tablespoon ≈ 15 mL; 1 cup ≈ 240 mL; 1 bowl ≈ 250-300 mL)<br><br>Besides eating fruit as dessert, have you considered incorporating fruit into your daily recipes to increase your intake?

			Create an Exercise Schedule<br><br>Block exercise time into your calendar and treat it like an important appointment. This helps establish consistency.<br><br>Share the Journey<br><br>Find an exercise partner to encourage each other, celebrate progress, and stay motivated.<br><br>Track Progress and Celebrate Achievements<br><br>Use a journal or fitness app to record your workouts. Reward yourself when you reach milestones.<br><br>Stay Positive<br><br>Remind yourself of the benefits of exercise, including improved health, better mood, and increased confidence.	

5	Although alcohol may seem to help you fall asleep, it actually makes your sleep lighter and affects your energy levels and work performance the next day. Many people think drinking helps them relax, but feeling tired, having headaches, or experiencing memory lapses the following day are often signs from your body. Drinking less alcohol not only reduces harm to your health but also helps you get genuine rest.	Changing Smoking Habits Takes Time!<br>Did you know that Traditional Chinese Medicine (TCM) acupuncture may help with smoking cessation?<br>According to TCM, long-term smoking can lead to excessive heat in the lungs and stomach, depletion of body fluids, and symptoms such as coughing, dry throat, and irritability.<br>Acupuncture stimulates specific points along the lung, stomach, and spleen meridians, as well as the Shenmen point, to regulate energy flow, clear lung heat, strengthen the digestive system, and reinforce the determination to quit smoking.<br>Ear acupuncture, in particular, may help reduce the brain's dependence on nicotine.<br>The Pok Oi Hospital Chinese Medicine Smoking Cessation Service is available free of charge. For more information, please visit:<br>https://cms.pokoi.org.hk/service_05	Remember the Healthy Lifestyle Goal We Set Together?<br><br>The goal is to complete 2.5 hours of moderate-intensity aerobic exercise every week!<br><br>The "135" Aerobic Exercise Principle<br>"1"<br>Exercise once every day to stimulate metabolism and gain the benefits of physical activity.<br><br>"3"<br>Exercise for at least 30 minutes each session. During the first 30 minutes of exercise, the body primarily uses stored carbohydrates for energy. Longer sessions maximize aerobic benefits.<br><br>"5"<br>Exercise five days per week. Research suggests that exercising three to five times weekly is ideal for improving and stabilizing blood glucose levels.<br><br>For individuals with diabetes, the best time to exercise is one to two hours after meals or at least two hours after taking medication. Avoid exercising on an empty stomach to help maintain stable blood sugar.	Fruit-Based Recipes<br>1) Fruit Salad<br><br>Ingredients: Watermelon, Honeydew melon, Strawberries, Mint leaves, Lemon juice<br><br>Method Cut the fruit into bite-sized pieces, combine in a bowl, add mint leaves and lemon juice, toss gently, and enjoy a refreshing summer salad.<br><br>2) Fruit-Glazed Chicken<br><br>Ingredients: Chicken thighs, Orange juice, Lemon juice, Honey, Minced garlic<br><br>Method Marinate the chicken in orange juice, lemon juice, and honey. Pan-fry until golden brown, add garlic, and cook thoroughly.<br><br>3) Frozen Fruit Yogurt<br>Ingredients: Plain yogurt, Mixed fruits (such as bananas and strawberries), Honey<br><br>Method Mix diced fruit with yogurt and honey. Freeze for several hours and enjoy a healthy frozen dessert.<br><br>We hope these recipes inspire you to enjoy more fruit every day. Feel free to

				share your own healthy fruit recipes with us!

6	Don't forget the healthy lifestyle goal we set together to cut down on drinking.<br>Fruits are rich in fiber, vitamins, and antioxidants. Eating more fruit can increase fullness, help you manage your diet while cutting down on alcohol, and reduce cravings for alcohol.	Struggling with Smoking Withdrawal Symptoms? Here Are Some Tips!<br>1) Difficulty Sleeping Nicotine withdrawal can disrupt sleep patterns. Try avoiding caffeinated and alcoholic beverages and engage in light exercise before bedtime.<br>2) Constipation Reduced nicotine intake can affect bowel movements. Eat more fruits and high-fiber foods such as whole grains, beans, broccoli, and bananas to promote healthy digestion.<br>3) Increased Appetite Choose low-calorie snacks such as high-fiber dried fruits, oat bars, baby carrots, sugar-free gum, mints, or fresh fruit. Chewing slowly can increase feelings of fullness and help prevent excessive calorie and fat intake during smoking cessation.<br>The earlier you quit smoking, the greater the benefits! Research shows that quitting before age 60 can recover up to three years of life expectancy lost due to smoking. Keep working toward your health goals!	Knee-Lifting Exercises: Simple and Effective Cardiovascular Training<br><br>According to the Department of Health, accumulating 150 minutes of moderate-intensity physical activity per week helps maintain cardiovascular health. Activities such as brisk walking, cycling, and stair climbing are excellent options.<br><br>Here are three knee-lift exercise variations:<br><br>1) Basic Knee Lift<br><br>Stand with feet shoulder-width apart and hands on hips. Alternately raise each knee as high as comfortable. Complete 8 repetitions.<br><br>2) Knee Lift with Arm Extension<br><br>Stand with feet shoulder-width apart and raise both arms overhead. Alternate lifting each knee while bringing the arms downward. Complete 8 repetitions.<br><br>3) Advanced Version<br><br>Raise your right arm overhead. Lift your left knee and gently touch it with your right hand. Perform 8 repetitions and switch sides.	Eye Health Starts with Your Diet: Vegetables Are Essential!<br><br>Did you know?<br><br>Dark leafy vegetables such as kale, spinach, and Chinese kale are rich in antioxidants and vitamins that help protect eye health and reduce the risk of vision deterioration.<br><br>According to healthy eating guidelines, adults should consume at least 3 servings of vegetables every day.<br><br>Benefits of Eating More Vegetables<br><br>Protect Vision May help reduce the risk of macular degeneration and cataracts as we age.<br><br>Reduce Eye Fatigue Vegetables rich in vitamins A and C may help reduce eyestrain, especially after prolonged screen use.<br><br>Support Overall Health Healthy eyes are closely linked to overall well-being and quality of life.<br><br>Try eating vegetables of at least three different colors daily to achieve a more balanced diet.

			Always warm up, stretch, and loosen tight muscles before exercising. If you experience discomfort during exercise, stop immediately and rest.	

7	Many beer companies in Germany and Japan now offer alcohol-free beers! Next time you're dining out with friends, why not give them a try?	Think Quitting Smoking Is All About Willpower? Think Again!<br>You've already made up your mind to quit, but certain moments, such as after finishing work or during stressful situations, suddenly make you want another cigarette?<br>These urges usually last only a few minutes. It's not because your body truly needs a cigarette, but because your brain has become accustomed to using smoking as a way to relax.<br>Successful quitting isn't just about willpower. It's about understanding why you want to change.<br>For example:<br>• Sleeping better<br>• No longer coughing<br>• Reducing worries for your family<br>• Improving your overall health<br>Next time a craving strikes, try this three-step method:<br>1) Take 15 deep breaths and ask yourself: "Do I really want a cigarette, or do I just want to relax?"<br>2) Drink water or have a mint. Give your mouth a different sensation.<br>3) Record your emotions at that moment, such as feeling tired, bored, or angry.<br>Over time, you may discover that you don't actually want a cigarette. Instead, you're trying to escape a particular feeling.	Gentle Daily Stretching Can Make a Difference<br><br>I recommend doing simple stretching exercises every day. Exercise doesn't have to be intense to be beneficial.<br><br>Simple stretching can:<br>Improve flexibility<br>Enhance circulation<br>Help you feel more relaxed<br>Suggested Stretches<br><br>1. Neck Stretch Tilt your head gently to one side and hold for 15-30 seconds. Repeat on the other side.<br>2. Shoulder Stretch Bring one arm across your chest and gently pull it closer using the opposite hand. Hold for 15-30 seconds and switch sides.<br>3. Back Stretch Clasp your hands together, stretch upward, then lean gently to each side. Hold for 15-30 seconds.<br>4. Quadriceps Stretch While standing, pull one ankle toward your buttocks and hold for 15-30 seconds. Repeat with the other leg.<br>5. Calf Stretch Face a wall, extend one leg behind you, and keep the heel on the ground. Hold for 15-30 seconds and switch sides.<br><br>Start slowly and progress gradually to help relax your body and mind.	An Easy Way to Eat More Fruits and Vegetables<br><br>Remember the healthy lifestyle goal we set together?<br><br>Eat 5 servings of fruits and vegetables every day!<br><br>It may seem difficult, but there are many simple ways to reach this goal without even noticing.<br><br>One easy method is to add fruits and vegetables while making soup and eat the ingredients along with the broth.<br><br>Why not try incorporating fruits and vegetables into your homemade soups and help your entire family build healthy habits?<br><br>For Individuals with High Blood Pressure<br><br>Excessive salt intake can contribute to fluid retention and high blood pressure.<br>1) Use less salt when cooking.<br>2) Reduce soy sauce and other high-sodium seasonings.<br>3) Consider switching to reduced-sodium soy sauce or oyster sauce.<br><br>These small changes can help support

				your cardiovascular health while making it easier to achieve your daily fruit and vegetable goals.

8	Want to drink less alcohol? Start by reducing your exposure to drinking environments, especially at parties or social gatherings. You can also add more ice to your drinks to reduce the amount of alcohol consumed!	Remember the healthy living goal we set together to quit smoking?<br>Acupressure Points That May Help You Quit Smoking<br>You can stimulate certain points on your hands and ears:<br>Hand Acupoints<br>• Lieque (LU7)<br>• Yangxi (LI5)<br>• Hegu (LI4)<br>Ear Acupoints<br>• Shenmen<br>• Stomach Point<br>• Heart Point<br>• Lung Point<br>• Endocrine Point<br>Why does this work?<br>Stimulating these acupoints may help reduce nicotine cravings and improve control over smoking urges. It may also help relieve withdrawal symptoms such as anxiety, irritability, and restlessness during the quitting process.	Nutrition Tips Before Exercise<br><br>Your food choices before exercise can have a significant impact on performance.<br><br>Carbohydrates Are Your Friend<br><br>Carbohydrates provide quick energy for physical activity.<br><br>Good options include:<br>Bananas: Rich in potassium and energy.<br>Oatmeal: Provides sustained energy.<br>Wholemeal bread: Pair with healthy toppings like peanut butter.<br>Include Some Protein<br><br>Protein helps maintain and repair muscles.<br>Suggested options:<br>Low-fat yogurt<br>Nuts<br>Stay Hydrated<br>Proper hydration is essential before exercising.<br>Drink water before activity to reduce the risk of dehydration. For particularly intense workouts, consider an electrolyte-containing beverage to replace minerals lost through sweating.	Remember the healthy lifestyle goal we set together? It's to eat 5 servings of fruits and vegetables every day!<br><br>Vegetables don't have to be eaten only during main meals.<br><br>Next time you're shopping for snacks, consider choosing vegetable-based options instead:<br><br>Dried vegetable snacks<br>Sweet potato fries or chips<br>Mushroom chips<br>Seaweed snacks<br>Mixed vegetable soup<br>Salad<br><br>These convenient choices make it easy to increase your vegetable intake anytime, anywhere. Just open the package and enjoy!<br><br>Vegetables contain antioxidants and a wide range of vitamins that help improve skin health and support immune function. They may also reduce the risk of cardiovascular disease, diabetes, and certain cancers.<br><br>When eating out, consider ordering an extra serving of vegetables!

9	Excessive alcohol consumption can raise pressure on blood vessel walls to unhealthy levels. Binge drinking can lead to long-term increases in blood pressure. Quit drinking now!	Why Does Your Skin Look Better After Quitting Smoking?<br>Many people notice that their skin appears brighter and healthier after quitting smoking, and it's not just their imagination.<br>Smoking causes blood vessels to constrict and reduces oxygen delivery to the skin. As a result, the complexion may become dull, yellowish, and prone to premature wrinkles.<br>After quitting smoking, blood circulation begins to improve within about 7 to 14 days.<br>The skin can "breathe" more easily again, leading to a healthier complexion and improved appearance.<br>Because you are no longer exposing your body to nicotine and other toxic substances, your body's natural repair processes can work more effectively, helping your skin recover more quickly.	Making Time for Exercise Even When You're Busy<br><br>We know your goal is to achieve 2.5 hours of moderate-intensity exercise every week.<br>"I'd love to exercise, but I just don't have time!"<br><br>Want simple exercises you can do at work or at home?<br><br>Check out the video above and follow along!<br><br>Doing the routine three times per day can help you achieve the weekly amount of exercise recommended for good health.<br><br>Regular exercise can:<br>Strengthen immunity<br>Reduce the risk of illness<br>Improve work productivity<br>Burn calories<br>Help maintain a healthy weight	Eating Out Often? Reaching Your Goal May Be Easier Than You Think!<br><br>A reminder of the healthy lifestyle goal we set together: eat 5 servings of fruits and vegetables every day.<br><br>If you're worried that eating out means you won't get enough fruits and vegetables, don't be! Achieving your health goal may be easier than you think.<br><br>During hot summer days, adding an extra serving of cold vegetable dishes can be both refreshing and a great way to work toward your daily 5-a-day goal.<br><br>When ordering drinks, consider choosing fruit toppings instead of pearls or other sweet add-ons. They provide more nutritional value while helping you increase your fruit intake.

10	Want to get rid of your beer belly? Excessive alcohol consumption is closely linked to the development of a beer belly, which not only affects your appearance but can also harm your health.<br>Alcohol is high in calories and promotes fat accumulation, especially around the abdomen. Time to cut down on drinking!	A Traditional Chinese Medicine Tea Recommended for Smoking Cessation<br><br>If you're trying to quit smoking, this herbal tea may be worth a try!<br><br>Houttuynia cordata is believed to help detoxify the body, support the elimination of harmful substances from tobacco, and improve lung inflammation. Licorice root, on the other hand, has soothing and calming properties that may help relieve anxiety and tension during the quitting process, helping you feel more relaxed.<br><br>Drinking 1 to 2 cups daily, especially when cravings strike, may help ease withdrawal symptoms and reduce the urge to smoke.<br><br>Additional benefits may include clearing heat, supporting detoxification, reducing inflammation, relieving swelling, promoting urination, and helping improve urinary tract inflammation. However, because it is considered slightly cooling in nature, it may not be suitable for individuals with a cold or deficient constitution.	Too Busy to Exercise? Think Again!<br><br>You may feel that busy schedules and office work leave no time for exercise.<br><br>In fact, 20 minutes of brisk walking or stair climbing each day can go a long way toward helping you achieve your weekly exercise target.<br><br>Try:<br><br>Getting off the bus or train one stop early.<br>Exiting the elevator one floor earlier and walking briskly to your destination.<br>Taking the stairs whenever possible.<br><br>Small changes can create meaningful health benefits over time.	Many people are probably planning festive meals with family and friends. Don't forget your health goal of eating 2 servings of fruit every day!<br><br>The following fruits are excellent choices after a festive feast and also carry auspicious meanings:<br><br>Pomelo The Chinese word for pomelo sounds similar to the word for "blessing" or "protection," symbolizing good fortune and family reunion. Pomelo also contains compounds similar to insulin, making it a beneficial fruit choice for people with diabetes.<br><br>However, individuals taking certain cardiovascular medications should consume pomelo with caution, as it may affect the body's ability to metabolize some medications.<br><br>Pear In Chinese culture, eating pears symbolizes "eliminating separation" and represents togetherness and reunion. Traditional Chinese Medicine considers pears cooling and hydrating, helping to nourish the lungs and relieve dryness, making them especially suitable during autumn.<br><br>Persimmon Persimmons symbolize "everything going smoothly" and are

				often associated with good luck. Their vibrant orange-red color also makes them an attractive addition to festive gatherings.<br><br>Star Fruit The Chinese pronunciation of "star fruit" resembles words associated with prosperity and success, symbolizing wealth, achievement, and a bright future.

11	Do you remember the healthy lifestyle goal we set together to help you reduce your alcohol intake?<br><br>The damage alcohol can cause to your liver should not be underestimated. Long-term drinking or alcohol abuse may lead to hepatitis and even liver cirrhosis. When you choose to drink, try reducing the amount and frequency so your liver can stay healthy and support a more vibrant life!	Are E-cigarettes Really Healthier?<br><br>Not necessarily!<br><br>Although e-cigarettes do not contain tar, they still contain nicotine, which is highly addictive, as well as other potentially harmful and cancer-causing substances. Therefore, e-cigarettes are not an effective way to quit smoking.<br><br>Quitting smoking doesn't have to happen overnight. Start with small daily changes, such as:<br><br>Putting away ashtrays<br>Not carrying a lighter<br>Avoiding smoking after meals<br><br>Start your quit-smoking plan today and make one small positive change each week!	Make the Most of the Holiday and Get Moving!<br>With less than two weeks until the Dragon Boat Festival and beautiful weather ahead, it's a great opportunity to get outdoors, not just enjoy festive food!<br><br>After typhoon season, autumn is an ideal time for hiking. Beginners can start with easy trails. The routes below can be completed in just 1 to 1.5 hours, offering beautiful scenery while helping you achieve nearly half of the recommended 150 minutes of weekly exercise.<br><br>Regular walking, hiking, and outdoor activities can:<br><br>Improve muscle and joint health<br>Strengthen the stability of the knees and ankles<br>Enhance muscle strength and coordination around the joints<br>Reduce stress and improve mood<br>Promote relaxation and overall well-being<br><br>Suggested beginner-friendly hiking routes:<br><br>Ho Pui Reservoir<br>Lai Chi Chong	remember to eat 3 servings of vegetables (about 1.5 bowls) each day to support digestion and regular bowel movements. Keep working toward your health goal!<br><br>Family hotpot gatherings are common during festive seasons, but did you know that the order in which you eat foods can make a difference?<br><br>1) Choose Protein Wisely<br><br>Seafood - Chicken - Pork/Beef<br><br>Seafood is a lean source of high-quality protein. Try to avoid high-fat meats such as marbled beef and fatty pork cuts.<br><br>2) Build a Nutritious Soup Base<br><br>Avoid deep-fried noodles and soups with excessive oil or added MSG.<br><br>Instead, use a variety of vegetables for your soup base, such as:<br><br>Radish<br>Seaweed<br>Mushrooms<br>Corn<br><br>This increases both nutrition and vegetable intake.

			Kiu Tsui Country Trail<br>Pineapple Dam Nature Trail	

12	Research shows that excessive alcohol consumption is associated with an increased risk of several cancers, including esophageal cancer and oral cancer. For your health and future, let's consider the possibility of quitting alcohol together. Even reducing your alcohol intake can significantly lower these risks.	Remember the healthy living goal we set together to quit smoking?<br>The First Step to Quitting Smoking Is Self-Awareness!<br><br>Start by observing your smoking habits:<br><br>At what times do you usually smoke?<br>How many cigarettes do you smoke each day?<br>Which day of the week do you tend to smoke more?<br><br>If you smoke more often during work breaks or leisure time, try finding activities that keep you engaged, such as mobile games or treating yourself to a favorite beverage.<br><br>If you struggle with cigarette cravings first thing in the morning, consider developing a new morning routine, such as drinking tea or coffee and going outside for some exercise and fresh air. The goal is to replace smoking as a source of dopamine release and relaxation.	Water Jogging: Exercise Without Knowing How to Swim!<br><br>Even if you can't swim, you can still enjoy water jogging!<br><br>Let's work together this month toward your health goals!<br><br>Swimming and Water Exercise May Help If You Have:<br>Joint Pain or Knee Pain<br>Swimming is a low-impact activity that places minimal stress on the joints. The buoyancy of water reduces pressure on the joints, making it an excellent option for people with arthritis or joint problems. Water also allows muscles and joints to move more freely.<br><br>Unstable Blood Pressure or Blood Sugar<br>Swimming strengthens cardiovascular endurance, improves circulation, and increases lung capacity. As an aerobic exercise, it can help lower blood pressure and cholesterol levels while strengthening the heart.<br><br>Back Pain or Frequent Fatigue<br>Swimming helps improve posture and balance. Water resistance strengthens the core muscles,	An Easy High-Fiber Recipe for Your Daily 3+2 Fruit and Vegetable Goal<br>Busy lifestyles often make it difficult to prepare vegetables regularly, but increasing fiber intake doesn't have to be complicated.<br>Adults and adolescents are recommended to consume at least 25 grams of dietary fiber daily.<br>Ingredients<br>1 cup rolled oats<br>2 cups water or milk (including plant-based milk)<br>½ cup green vegetables (such as spinach or kale)<br>¼ cup chopped carrots<br>¼ cup nuts (such as almonds or walnuts)<br>1 sliced banana<br>Honey or maple syrup (optional)<br>Preparation<br><br>Add fresh fruits such as banana slices, blueberries, or apple slices to your oatmeal for extra fiber and flavor.<br>If possible, prepare nuts as a snack. A handful of almonds or walnuts is an easy way to boost your daily fiber intake.<br><br>This simple and nutritious recipe can help you achieve your daily fruit and vegetable target more easily.

			promoting better body alignment and helping reduce back and spinal problems. Regular swimming may also boost immune function and improve overall resistance to illness.	

13	Long-term heavy drinking (more than 5 drinks per occasion) can lead to depression and anxiety, making it harder to cope with stress. This is because frequent heavy drinking disrupts the brain chemicals necessary for good mental health and normal functioning.<br>As the saying goes, "Drinking to drown your sorrows only creates more sorrow!"<br><br>Besides drinking, there are more effective ways to manage stress, such as exercise. Even a brisk walk can help clear your mind and reduce stress.	Cigarettes and Coffee, A Difficult Combination to Break!<br><br>If you enjoy caffeinated or carbonated beverages, this message may be especially relevant to you.<br><br>Caffeine stimulates the central nervous system and can be habit-forming. It may also increase the desire to smoke and make cigarette cravings more intense. As a result, newly quit smokers may be more tempted to smoke after consuming caffeinated drinks.<br><br>Nicotine suppresses some of caffeine's effects. During smoking cessation, continued caffeine consumption may make withdrawal symptoms feel more noticeable. In addition, caffeine has a diuretic effect that can increase thirst, which may trigger smoking urges.<br><br>Adjusting your dietary habits early can make your journey to becoming smoke-free much easier.	Feeling Stuffed After the Holidays? Follow this simple workout during your lunch break and spend just 5-10 minutes moving to help burn abdominal fat.<br><br>Core-strengthening exercises not only help train your abdominal muscles but also promote proper posture, especially if you spend long hours sitting or standing. This may help reduce back pain and discomfort.<br><br>Want a simple way to stay active and work toward your weekly 150-minute exercise goal?<br><br>Joining group exercise classes is a great option! Not only can you meet new friends, but you can also learn proper techniques under professional guidance.<br><br>Whether it's yoga, dance, or fitness training, choose an activity you enjoy and make exercise more fun.<br><br>Some organizations offering regular exercise programs include:<br><br>▫ Hong Kong Federation of Trade Unions Continuing Education Centre<br><br>▫ YMCA Hong Kong	Vegetables Are Everywhere!<br><br>You may not realize it, but vegetables can be added to almost every meal. With just one extra step, your goal of 3 servings of vegetables per day can be achieved without much effort.<br><br>Add corn kernels or mushrooms while cooking rice. For added convenience, use canned vegetables that require no washing.<br><br>When choosing dumplings or dim sum, select vegetable-rich options such as vegetable dumplings, cabbage dumplings, or chive dumplings.<br><br>Use a blender to prepare vegetable and fruit smoothies for breakfast as an alternative to high-carbohydrate breakfasts. This can help stabilize blood sugar and support gut health.<br><br>Add vegetables such as spinach or corn when preparing egg dishes. They're delicious and suitable for the whole family.

			▫ Leisure and Cultural Services Department	

14	Watching sports without alcohol! Alcohol is carcinogenic and high in calories.<br><br>Whether it's beer, alcopops, red wine, white wine, or other alcoholic beverages, there is no completely safe level of alcohol consumption, and drinking can harm your health!	Smoking Doesn't Just Harm Your Lungs, It Can Harm Your Eyes Too<br><br>While most people know that smoking damages the lungs, fewer realize that it can also affect eye health.<br><br>Toxic chemicals in cigarette smoke enter the bloodstream and can damage sensitive tissues in the retina, lens, and macula.<br><br>The most effective way to reduce the risk of smoking-related eye diseases is to quit smoking completely.<br><br>No matter your age, quitting smoking can help slow the progression of eye diseases and reduce the risk of age-related macular degeneration and glaucoma.<br><br>As the holiday season approaches, enjoy more healthy social activities and fewer cigarettes to make your celebrations even more enjoyable.	How Is Your Weekly Exercise Goal Going?<br><br>Spending a little time exercising every day can help you build a lasting habit and ease muscle soreness.<br><br>Exercise Fact: Is It Better to Exercise Before or After Meals?<br><br>Research suggests that exercising before meals can improve insulin sensitivity, enhance blood glucose uptake, and may have greater fat-burning benefits than exercising after meals.<br><br>Another advantage is flexibility, as pre-meal exercise is less restricted by meal timing.<br><br>If you prefer exercising after meals, it can help your body use blood glucose as energy and reduce post-meal blood sugar spikes.<br><br>However, it is recommended to wait at least one hour after eating before engaging in moderate-intensity exercise to avoid digestive discomfort.	How Can You Strengthen Your Immune System During Flu Season?<br><br>Besides getting enough sleep, eating 5 servings of fruits and vegetables every day is one of the most natural ways to support your immune system.<br><br>One serving is approximately the size of your fist.<br><br>Dark green and orange-colored fruits and vegetables are rich in vitamin A, which helps protect the lining of the respiratory tract.<br><br>Citrus fruits, guava, and kiwifruit are rich in vitamin C, which supports immune function.<br><br>The more colorful your fruits and vegetables, the broader the range of nutrients you'll receive.<br><br>Try eating 5 servings of fruits and vegetables every day for five consecutive days. You may notice:<br><br>Better energy levels<br>Healthier-looking skin<br>Reduced allergy symptoms

15	Are alcopops really as harmless as they look?<br><br>"Alcopops" are alcoholic beverages mixed with fruit juice, flavorings, and sometimes caffeine.<br><br>Although their alcohol content may be similar to beer (around 4-7%), some varieties can contain as much as 8-12% alcohol.<br><br>Their sweet, fruity taste can mask the alcohol flavor, making it easy to drink more than intended and increasing the risk of binge drinking and related health problems.	Have You Considered How Long-Term Smoking Affects Your Sleep?<br><br>Smoking is associated with many physical and mental health conditions, including cancer, heart disease, obesity, and mental health disorders. It can also negatively affect sleep quality.<br><br>Research shows that long-term smokers are twice as likely to experience sleep problems compared with non-smokers. Common issues include difficulty falling asleep and excessive daytime sleepiness.<br><br>Long-term smokers also tend to spend less time in deep sleep and generally experience poorer sleep quality.<br>Will you sleep better immediately after quitting smoking?<br>Not necessarily.<br>Withdrawal symptoms can also affect sleep. During the early stages of nicotine withdrawal, people may wake up more frequently during the night and feel that their sleep quality has worsened.<br><br>However, as the withdrawal period progresses, daytime sleepiness gradually decreases and sleep quality may improve.<br>How can you minimize the impact of	Improving Pain Through Simple Rehabilitation Exercises<br><br>If you'd like to reduce pain and discomfort, you can try rehabilitation exercises at home to strengthen your lower back and side abdominal muscles.<br><br>Just 20 minutes a day, whether at home or during work breaks, can help you work toward your health goals.<br><br>For individuals with chronic lower back pain:<br><br>Avoid prolonged sitting → promotes better blood circulation<br><br>Engage in low-impact aerobic exercise regularly, such as walking, swimming, or yoga → strengthens core muscles and improves stability<br><br>When sleeping on your side, place a pillow between your knees → helps reduce pressure on the lower back<br><br>Stretching tight muscles before engaging in regular exercise can improve effectiveness and reduce the risk of injury.	Don't Like Fruit? Vegetables Alone May Not Be Enough!<br><br>Your daily health goal is 5 servings of fruits and vegetables.<br><br>The more colorful your produce choices, the more likely you are to obtain a wide range of vitamins, minerals, and fiber.<br><br>If you don't enjoy eating fruit but still want to get enough nutrients, consider these ideas:<br><br>Add fruit to tea or sugar-free sparkling water for a refreshing, low-calorie drink.<br><br>Turn fruit juice into homemade ice pops and add pieces of fresh fruit.<br><br>If you're busy at work or school, try:<br><br>100% pure fruit juice<br>A small serving of grapes<br>Cherry tomatoes<br><br>These options make it easier to increase your fruit intake and stay on track with your healthy eating goals.

		withdrawal on sleep?<br>Maintain a regular exercise routine. Exercise stimulates the release of endorphins, helping you feel relaxed, improving mood, and reducing the discomfort associated with withdrawal.<br>Let's quit smoking together and set higher expectations and healthier goals for the new year!		

16	Have you continued reducing the frequency and amount of alcohol consumption this month?<br><br>Did you know that drinking alcohol not only harms the liver, but can also deplete important nutrients in your body?<br><br>Research has shown that long-term alcohol consumption can significantly reduce levels of B vitamins, particularly vitamin B1 (thiamine), folate (vitamin B9), and vitamin B12. Deficiencies in these essential nutrients may lead to serious neurological damage and increase the risk of cognitive decline, dementia, and alcohol-related brain disorders.<br><br>Reducing alcohol intake not only benefits your liver, but also helps your body maintain the nutrients needed to support a healthy brain and nervous system. Keep up the good work as you	Smoking May Age Your Brain and Cognitive Function by 10 Years<br><br>Do you remember the goal we set at the start of your health program: Quit Smoking?<br><br>Nicotine stimulates the release of dopamine, creating pleasurable feelings. However, over time, smoking may contribute to continued decline in memory and cognitive function, regardless of how frequently a person smokes.<br><br>When cravings strike:<br><br>Find something to replace smoking and keep your mouth or hands occupied.<br><br>While driving, waiting for transport, or eating at a restaurant, sugar-free chewing gum can satisfy oral cravings without adding extra calories.<br><br>While watching television or using your phone, squeezing a stress ball can be a useful distraction. It not only helps shift your attention away from cigarettes but also relieves stress. Keeping your hands occupied can make it easier to resist smoking urges.	Improve Your Physique Without Breaking a Sweat!<br><br>Do you often plan to exercise but feel too tired after a full day of work?<br><br>This week, why not try beginner-level Pilates?<br><br>Pilates can help:<br><br>Relax tight muscles<br>Improve posture<br>Enhance flexibility<br>Build core strength<br><br>Following this video for 10 minutes every morning and evening can add up significantly toward your recommended 150 minutes of weekly exercise.<br><br>Pilates may also help relieve common problems experienced by office workers, including:<br><br>Forward head posture ("tech neck")<br><br>Repetitive strain from mouse use<br><br>Frozen shoulder and shoulder discomfort<br><br>Remember to maintain balance between your left and right sides and	No matter how busy work gets, before placing your order, take a moment to ask yourself whether you've achieved your health goal of 5 servings of fruits and vegetables today.<br><br>Swap your side dish for vegetables<br>For example, at McDonald's or KFC, choose a salad, corn cup, or mashed potatoes instead of fries. At Subway, you can add extra vegetables at no additional cost!<br><br>Bring your own vegetables<br>When buying takeaway, consider bringing a ready-to-eat salad cup, cherry tomatoes, or mini cucumbers and enjoy them alongside your meal.<br><br>Choose healthier drinks<br>Opt for fruit tea, lemon tea, or freshly squeezed fruit juice instead of sugary beverages.

	work toward your health goals!		focus on muscle coordination to avoid placing excessive strain on one side of the body.	

17	Alcohol abuse can seriously damage the body, increasing the risk of ulcers, diabetes, cancer, sexual dysfunction, liver disease, and heart disease.<br><br>Is a brief period of pleasure really worth potentially irreversible harm?<br><br>Happiness can also come from many healthier activities, such as exercising, spending quality time with family and friends, and maintaining good health.	Can Smoking Cause Blood Vessel Blockages?<br><br>Although the exact cause is not fully understood, tobacco use has been strongly linked to Buerger's disease, and nearly all people diagnosed with the condition are smokers or users of other tobacco products, including King George VI of the United Kingdom.<br><br>Chemicals in tobacco irritate the lining of blood vessels, causing inflammation and swelling. This can reduce blood flow, obstruct blood vessels, and increase the risk of blood clot formation.<br><br>Common symptoms include:<br><br>Pale, red, or bluish-purple fingers and toes (Raynaud's phenomenon)<br>Cold hands or feet, sometimes accompanied by pain or burning sensations<br>Pain in the legs or ankles while walking, often located around the arch of the foot<br>Painful skin ulcers on fingers or toes and, in severe cases, tissue death (gangrene)<br>The condition most commonly affects people between the ages of 20 and 45, and it is more common in males than females.	What Music Do You Listen to While Exercising?<br><br>You might think that soft, relaxing music makes exercise easier, but surprisingly, music with a stronger rhythm may improve workout performance and even enhance fat-burning efficiency!<br><br>Studies suggest that listening to music during exercise can:<br><br>Extend the duration of aerobic workouts<br>Improve glucose utilization<br>Enhance blood sugar control<br>Indirectly improve fat metabolism<br><br>Steady-tempo music<br><br>Helps maintain a consistent pace<br>Reduces perceived effort<br>Makes exercise feel easier and more sustainable<br><br>Irregular-tempo music<br><br>May disrupt breathing rhythm and pacing<br>Can increase feelings of fatigue<br><br>Create your own workout playlist today and stay motivated to achieve	Did You Know Vitamin C Does More Than Boost Immunity?<br>1) Essential for Collagen Production<br><br>Vitamin C is required for the synthesis of collagen, a major component of joint cartilage. Collagen helps maintain joint flexibility and stability.<br><br>2) A Powerful Antioxidant<br><br>Vitamin C helps neutralize free radicals and reduce oxidative stress. Excessive oxidative stress can contribute to inflammation and joint damage, increasing the risk of joint disorders.<br><br>3) Supports Calcium Absorption<br><br>Vitamin C helps the body absorb and utilize calcium more effectively. Adequate calcium intake plays an important role in preventing osteoporosis and maintaining healthy bones and joints.<br><br>How much is enough?<br>According to the U.S. Dietary Reference Intake (DRI), the recommended daily intake is:<br>75 mg for women<br>90 mg for men<br>One medium-sized orange or kiwifruit already provides approximately 70 mg

		Let's quit smoking together and reduce the risk of cardiovascular disease!	your weekly 150-minute exercise goal!	of vitamin C!<br><br>Remember, eating fruits and vegetables can help support joint health, so keep working toward your 5 servings of fruits and vegetables each day!

18	Not drinking alcohol? What else can you choose?<br><br>Explore alcohol-free beverage options in Hong Kong:<br><br>Sparkling tea<br>Soda water<br>Functional sparkling drinks<br>Alcohol-free lager<br>Alcohol-free distilled spirits<br>Alcohol-free sparkling wine	Did You Know You're Inhaling More Than Just Nicotine and Tar?<br><br>Traditional cigarettes contain over 7,000 harmful chemicals, more than 70 of which are known carcinogens.<br><br>Carbon monoxide is particularly harmful because it directly affects brain function, intelligence, and memory.<br><br>According to the World Health Organization, one out of every two long-term smokers dies prematurely due to smoking, losing an average of 15 years of life expectancy.<br><br>The good news is that your body begins to recover as soon as you stop smoking:<br><br>Within 12 hours: Carbon monoxide levels in the blood return to normal.<br><br>Within 5-15 years: The risk of stroke drops to a level comparable to that of a non-smoker.<br><br>Take action today:<br>1) Tell your friends and colleagues that you are quitting smoking so that they can support and encourage you.<br>2) Use evidence-based smoking cessation methods or professional	Walking 10,000 Steps a Day Isn't Necessarily Healthier!<br><br>Research from the United Kingdom suggests that the key to better health isn't simply the total number of steps you take each day, but the intensity of your walking.<br><br>Slow walking at a pace of fewer than 60 steps per minute may actually be associated with a higher risk of knee osteoarthritis, as low-intensity walking can lead to repeated stress and friction on the knee joints.<br><br>In contrast, brisk walking or light jogging at a pace of 60 steps per minute or more may significantly reduce the risk of developing knee osteoarthritis.<br><br>Research findings include:<br><br>4,000-5,999 steps per day - 16% lower risk of knee osteoarthritis<br>8,000+ steps per day - 26% lower risk of knee osteoarthritis<br><br>In addition, activities that increase your heart rate help improve cardiovascular fitness and contribute toward the recommended 150 minutes of exercise per week.	Many people struggle to eat enough fruits and vegetables and may wonder whether juice can be a substitute. But how does juice compare with whole fruits and vegetables?<br><br>1) Loss of Dietary Fiber<br>When fruits or vegetables are juiced, only about 10-20% of the original fiber remains.<br><br>To retain more fiber, avoid straining out the pulp. Keeping the pulp can preserve over 90% of the dietary fiber.<br><br>2) High Sugar Content<br>Many commercial fruit juices contain up to 25 grams of sugar per serving.<br><br>Making your own juice allows you to:<br><br>Avoid added sugar<br>Control the ratio of fruits and vegetables<br>Improve nutritional value<br>3) Reduced Satiety<br>Juice generally provides less fullness compared with whole fruits and vegetables.<br><br>To slow digestion and improve satiety, consider adding healthy fats or protein sources such as avocado.<br>While juice can be a helpful supplement, it should not completely

		services rather than switching to another tobacco product.<br>3) If needed, call the Department of Health's Integrated Smoking Cessation Hotline: 1833 183.	A brisk 20-minute walk during your commute or on your way home can make a meaningful difference!	replace fresh fruits and vegetables. Remember: your daily goal is 5 servings of fruits and vegetables!

19	What's the difference between drinking alcohol on an empty stomach and drinking after a meal?<br><br>Drinking on an empty stomach can easily cause hypoglycemia (low blood sugar). Alcohol suppresses glucose production, inhibits glycogen breakdown, and reduces insulin resistance, potentially leading to low blood sugar. Even small amounts of alcohol can trigger this effect. Therefore, avoiding alcohol on an empty stomach is especially important for people with diabetes.<br><br>Drinking after a meal can lead to high blood sugar. Unlike drinking on an empty stomach, alcohol consumed after eating can increase insulin resistance and stimulate glycogen breakdown, causing post-meal blood sugar elevations. For people with poorly controlled diabetes, especially those	Noticed Your Hairline Moving Backward Recently?<br><br>Smoking may be one of the reasons!<br><br>Research suggests that nicotine can damage hair follicles by causing blood vessels to constrict, reducing the supply of nutrients and oxygen to the follicles. It may also trigger inflammation, which can worsen hair loss.<br><br>Harmful chemicals in cigarette smoke generate free radicals that damage cells, accelerate hair aging, and contribute to hair shedding.<br><br>Want to protect your hair and avoid premature hair loss? Quitting smoking could give your hair a better chance to thrive!	How Can Outdoor Fitness Equipment Help You Stay Active Every Day?<br><br>Stationary Bike: Primarily used for aerobic exercise and lower-body strength training. Whether you choose a steady pace or interval cycling, it can help improve cardiovascular fitness. Aim for 10 minutes per session.<br><br>Pull-up Bar: Helps develop upper-body strength and flexibility, strengthening the arms and shoulders while promoting blood circulation. Perform 10-15 repetitions, repeating for 2-3 sets.<br><br>Elliptical Walker: Provides a full-body workout, particularly for the legs and core muscles, while helping improve balance. Aim for 10 minutes per session.<br><br>Stepper: Hold onto the handrails, place one foot on the upper pedal and the other on the lower pedal, then alternate in a stair-climbing motion. Aim for 10 minutes per session.<br><br>Whether you're on your lunch break or waiting for takeout, there's probably a park nearby. Let's work together toward your goal of 150 minutes of exercise every week!	If there's a perfect timing for work, is there also a perfect time to eat fruit?<br><br>Before Meals<br><br>Eating fruit before meals may:<br><br>Stimulate digestive juices<br>Aid digestion<br>Improve the absorption of vitamins and minerals<br><br>For those trying to lose weight, the fiber and water content in fruit can increase feelings of fullness and help reduce overall food intake.<br><br>However, individuals with sensitive stomachs should avoid eating high-fiber fruits on an empty stomach.<br><br>After Meals<br><br>Eating fruit after a meal may help digestion because fruit contains various natural enzymes.<br><br>The protein, healthy fats, and fiber from the main meal can also slow digestion, allowing fruit sugars to be released more gradually and helping maintain stable blood sugar levels.<br><br>Take action now and think about which fruit you can add to tonight's

	at high risk of diabetic emergencies, this may increase the risk of diabetic ketoacidosis.			meal to help achieve your daily 5 servings of fruits and vegetables!

20	Excessive Alcohol Consumption Affect Your Vision<br><br>Alcohol has a depressant effect on the brain and can interfere with nerve transmission. The optic nerve, which plays a crucial role in vision, is one of the nerves that can be affected by alcohol. Long-term excessive drinking may gradually damage these nerves, causing vision to become increasingly blurred.<br><br>The liver is responsible for many of the body's metabolic functions. When excessive alcohol intake impairs liver function, the metabolism of vitamin A, an essential nutrient for eye health, may be affected. This can lead to problems such as night blindness and blurred vision. In addition, a deficiency of vitamin B1 (thiamine) may result in weakening or paralysis of the eye muscles.	Is Smoking Related to Gum Disease?<br><br>Damages the Oral Environment<br><br>Nicotine weakens the immune system and reduces the body's ability to fight dental plaque. This allows harmful bacteria to damage gum tissues more easily, increasing the risk of tooth decay and periodontal disease.<br><br>Harms Dental Health<br><br>Smoking doesn't just contribute to gum disease. Tar buildup can stain teeth yellow and black, cause bad breath, and damage the supporting bone structure around teeth. The longer you smoke, the greater the impact on periodontal health.<br><br>Masks Early Warning Signs<br><br>Nicotine causes small blood vessels to constrict, reducing blood flow to the gums. As a result, gums may appear pale rather than red and swollen, making early symptoms harder to detect. Many people delay seeking treatment until teeth become loose or gums recede, by which time periodontal disease may already be severe.<br><br>Quit smoking today and maintain good	Isn't Gaming Just Sitting Around?<br><br>Not necessarily! There are many active video games that can help you achieve your daily 20 minutes of exercise without leaving home.<br><br>Examples include:<br><br>Ring Fit Adventure (Nintendo Switch)<br>Just Dance<br>Mario Party<br><br>You can even work toward your health goals with friends and family.<br><br>No game console at home? Don't worry! Here are some simple and fun alternatives:<br><br>Play table tennis across the dining table<br><br>Use a self-rebounding badminton trainer and play solo<br><br>Follow online dance tutorials and get moving while having fun<br><br>Exercise can be enjoyable, no matter where you are!	A baked orange can be a warming and comforting snack.<br><br>Method:<br><br>Wash the orange thoroughly.<br>Wrap it (with the peel on) in aluminum foil.<br>Bake in a preheated oven for approximately 5-10 minutes.<br>Remove once fragrant.<br>Serve with preserved plum if desired.<br><br>Orange peel can also be used to make a warm tea.<br><br>Simply:<br><br>Wash the orange peel thoroughly.<br>Add slices of ginger and a small amount of brown sugar.<br>Pour in hot water and allow it to steep.<br><br>Enjoy a comforting citrus tea!

	Excessive alcohol consumption can also cause blood vessels to dilate, potentially damaging the retina. For people with conditions such as high blood pressure or diabetes, this may increase the risk of retinal bleeding and other eye complications.<br><br>That's why alcohol should be consumed in moderation and not as a regular habit. Why not start taking steps today to reduce your alcohol intake and protect both your vision and overall health?	oral hygiene habits to protect your own health and that of your family and friends.		

21	Does drinking alcohol make people less sharp?<br><br>1) Alcohol can damage the brain.<br>2) Alcohol is toxic to the central nervous system, affecting judgment and balance, increasing the risk of falls, accidents, and brain injury.<br>3) It can impair metabolism, heart function, and blood supply, contributing to malnutrition.<br>4) It interferes with the absorption of vitamin B1 (thiamine), an essential nutrient for brain health.	How Does Smoking Affect Bone Health?<br><br>Nicotine and the heavy metal cadmium have direct toxic effects on bone cells. They reduce and interfere with the body's ability to absorb calcium and vitamin D, increasing the risk of osteoporosis.<br><br>Smoking can also reduce the beneficial effects of estrogen and may lead to menopause occurring one to four years earlier, accelerating calcium loss. Women are particularly vulnerable to osteoporosis after menopause.<br><br>Research shows that smokers have an 80% higher risk of hip fracture than non-smokers.<br><br>Want to stay active and maintain strong bones as you age? Quitting smoking is one of the best things you can do for your bone health.	Why Is Core Training So Important?<br><br>The core is more than just your abdominal muscles. It includes a large group of muscles involving the abdomen, lower back, pelvic floor, and waist.<br><br>Benefits of strengthening your core include:<br><br>1) Improved posture<br>Less slouching<br>Better body alignment<br>2) Reduced lower back pain<br>Helps support the spine<br>Distributes stress more effectively<br>3) Enhanced balance and athletic performance<br>Makes daily activities safer and easier<br><br>One of the most noticeable benefits is a flatter-looking waistline. As your core becomes stronger, your midsection naturally appears more toned.<br><br>Remember to maintain steady breathing throughout your workout to train both your muscles and cardiovascular system.	Research from the United States has found that levels of vitamin B2, vitamin C, and vitamin E remain largely unchanged after freezing. In some cases, certain vitamin levels may even increase after freezing.<br>Only beta-carotene levels in vegetables such as carrots, spinach, and peas may decline more noticeably.<br><br>What's the Real Cause of Nutrient Loss?<br>In fact, nutrients are more likely to be lost during food preparation and cooking, particularly through boiling. Boiling can reduce nutrient levels by approximately 50-60%, especially water-soluble vitamins such as:<br>Vitamin B, Vitamin C<br><br>The longer vegetables are cooked, the greater the nutrient loss.<br>To preserve more nutrients:<br>Use less water<br>Steam vegetables briefly<br>Use a microwave for short cooking times<br><br>These cooking methods help retain more water-soluble vitamins and antioxidants.<br>Practical Tip:<br>If you don't have time to cook regularly, keep a bag of frozen vegetables in your freezer. You can

			Just 20 minutes of exercise each day can help you tackle everyday tasks more comfortably.	easily add them to noodles, soups, or other meals and work toward your goal of 5 servings of fruits and vegetables every day!

22	Want to quit drinking but don't know where to start? Worried about withdrawal symptoms?<br><br>You can search your mobile app store for alcohol reduction or sobriety apps that support a healthier alcohol-free lifestyle.<br><br>You can also: 1) Record your drinking habits, including spending and alcohol consumption.<br>2) Track your sleep, energy levels, mood changes, and cravings.<br>3) Keep a journal of your thoughts, feelings, and daily reflections to stay motivated.<br>4) Monitor your current sobriety streak and personal best record.	Is Smoking Your Way of Managing Emotions?<br><br>Do you find it difficult to quit because you're bored at work or feeling stressed?<br><br>The next time you want a cigarette, ask yourself:<br><br>"What else can I do right now?"<br><br>Try this simple conversation with yourself when cravings arise:<br><br>30-Second Practice<br><br>1) Pause: Before lighting a cigarette, stop for 10 seconds.<br><br>2) Delay for 10 minutes: Tell yourself, "I'm not allowed to smoke for the next 10 minutes."<br><br>3) Choose a replacement activity for 2 minutes:<br><br>Slowly drink a large sip of water.<br>Take 10 deep breaths (inhale for 4 seconds, exhale for 6 seconds).<br>Chew gum or suck on a mint.<br>4) Ask yourself: "What emotion am I trying to deal with right now?"<br>You'll often find that cravings come in waves. Delaying and using	Did You Know Singing Can Be a Form of Aerobic Exercise?<br><br>Besides helping you relax, singing can actually provide a mild aerobic workout!<br><br>Although singing seems to involve only the mouth and voice, it engages muscles throughout the body.<br>Singing can burn approximately 2 kilocalories per minute, especially when you are breathing deeply, using your abdominal muscles, and feeling slightly out of breath.<br><br>You may not realize it, but singing loudly can also increase your heart rate, producing effects similar to light aerobic exercise.<br><br>For people with poorer circulation, the deep breathing and abdominal muscle engagement involved in singing can:<br><br>Improve blood circulation<br>Support heart health<br>Help reduce snoring<br>Improve symptoms associated with sleep apnea<br><br>While singing alone is unlikely to help you reach the recommended 150 minutes of weekly exercise, it offers	Healthy joints are important not just for people who enjoy sports. Everyday activities such as climbing stairs or getting up from a chair also rely heavily on healthy joints.<br><br>Research has shown that, alongside exercise, collagen supplementation may help improve joint and tendon health. However, collagen works best when combined with adequate vitamin C intake.<br><br>Vitamin C is essential for collagen synthesis.<br><br>Consuming 5-15 grams of collagen daily for at least three months may support healthy joints and tendons.<br><br>At Holiday Gatherings, These Foods Are Rich in Vitamin C:<br><br>Fruit platters, especially:<br><br>Kiwifruit<br>Strawberries<br>Oranges<br><br>Roasted vegetables, such as:<br><br>Red bell peppers<br>Green bell peppers<br><br>Tomatoes, whether in salads or side

		replacement activities can help the urge pass naturally.	many health benefits and may make other forms of exercise easier and more enjoyable.	dishes<br><br>Combining collagen with vitamin C-rich foods may help maximize the benefits for joint health.

23	Remember the healthy lifestyle goal we set together to cut down on drinking?<br>There are several ways to begin cutting down on alcohol:<br><br>1) Drink smaller servings, such as replacing one large beer with two small beers.<br>2) Choose drinks with lower alcohol content. Paying attention to alcohol percentage is a good first step.<br>3) For every alcoholic drink, have a glass of water, a non-alcoholic beverage, or a snack.	"I've Failed to Quit Smoking Before. Can I Still Succeed?" Absolutely.<br><br>Many people who relapse after quitting believe they simply lack willpower.<br>In reality, a relapse is not a permanent failure. Every relapse is an opportunity to understand your triggers more clearly. Was it stress? Low mood? Being around friends who smoke?<br><br>More than half of successful quitters have relapsed at least once before eventually succeeding.<br>Once you understand what led to the relapse, you'll be better equipped to respond differently the next time.<br>Hong Kong Civil Service health guidelines recommend:<br>Setting a quit date<br>Seeking support<br>Tracking your progress<br>Preparing strategies to overcome obstacles<br><br>Start by identifying the situations in which you're most likely to smoke, such as:<br>After meals<br>When stressed<br>While drinking alcohol<br>When meeting certain friends<br><br>Then prepare an alternative action for	Relieve Neck, Shoulder, and Back Tension from Office Work<br><br>Long hours spent using a computer mouse or looking down at a smartphone can lead to:<br><br>Neck and shoulder stiffness<br>Lower back pain<br>Muscle tightness<br><br>If left unmanaged, these issues may contribute to carpal tunnel syndrome, resulting in numbness, tingling, and discomfort in the wrists and fingers, which can affect both work and quality of life.<br><br>Did you know that when your head tilts forward at a 45-degree angle, the load placed on your cervical spine becomes more than four times greater than when your head is upright?<br><br>Follow this stretching routine to loosen up those tight muscles!<br><br>Once you've relaxed and stretched your muscles, combining stretching with regular exercise can make it easier to stay active and achieve your weekly exercise goals.	Fruits Can Help You Sleep Better Too!<br><br>Eating fruit isn't just good for your physical health. Certain fruits have calming and nourishing properties that may help improve sleep quality.<br><br>Remember your goal of 5 servings of fruits and vegetables every day, and continue building healthier habits for the year ahead!<br><br>Cherries<br><br>Contain natural melatonin, which helps regulate the body's sleep-wake cycle.<br><br>Best time: 1-2 hours before bedtime<br>Recommended amount: 10-15 fresh cherries<br><br>Bananas<br><br>Help relax muscles and calm the nervous system.<br><br>Best time: After dinner or about 30 minutes before bedtime<br><br>For extra relaxation, enjoy with warm milk or longan and red date tea.<br><br>Kiwifruit<br><br>May improve sleep quality, provide

		each situation. A little planning can make quitting much more effective.		antioxidants, and help you fall asleep faster.<br><br>Best time: About 1 hour after dinner<br><br>Avoid eating on an empty stomach.<br><br>Grapes<br><br>Contain melatonin and may help regulate sleep cycles and reduce difficulty falling asleep.<br><br>Best time: 1-2 hours before bedtime<br><br>Avoid eating them too late at night to prevent digestive discomfort.<br><br>Pears<br><br>Rich in water and B vitamins, which may help calm the nervous system.<br><br>Best time: About 1 hour after dinner<br><br>Avoid eating immediately before bedtime, as the high water content may lead to nighttime urination and disturb sleep.

24	What are the benefits of drinking less alcohol?<br><br>Some of the fastest noticeable benefits include:<br><br>Feeling more refreshed in the morning<br>Having more energy throughout the day<br>Improved skin condition<br>Greater motivation and productivity<br>Better weight management	Remember the healthy living goal we set together to quit smoking?<br><br>World No Tobacco Day<br><br>The World Health Organization designates 31 May each year as World No Tobacco Day, encouraging smokers to experience life without tobacco.<br><br>This year's theme is:<br><br>"Unmasking the Appeal: Exposing Industry Tactics on Tobacco and Nicotine Products."<br><br>One technique highlighted is the 4-7-8 Breathing Method, which can help distract your mind when cravings occur. Research suggests that smoking urges are often linked to stress and emotional responses, and controlled breathing can help regulate emotions and reduce immediate urges to smoke.<br><br>How to Practice the 4-7-8 Breathing Method<br>Step 1: Place the tip of your tongue against the roof of your mouth and exhale completely.<br>Step 2: Slowly inhale through your nose while counting to 4, then hold your breath for 7 seconds.	Yes, You Can Build Strength and Balance Without Breaking a Sweat<br><br>Studies have shown that people who used short breaks to exercise for just 10 minutes per day increased their muscle mass by an average of 0.42 kg, while trunk extension strength improved by 33%.<br><br>Try performing each exercise for 30-60 seconds, aiming for as many repetitions as possible, with minimal rest between exercises.<br><br>Bodyweight Squats Strengthen the legs and glutes. Simply lower yourself into a sitting position and stand back up.<br><br>Desk Push-ups Place your hands on the edge of a desk and perform push-ups to strengthen your chest and arms.<br><br>Lunges Step forward with one leg and lower your body to build balance, stability, and core strength.<br><br>Calf Raises Hold the back of a chair and lift your heels off the ground to improve lower-limb circulation and calf strength.<br><br>Wall Sits Lean against a wall and	Do you remember the goal we set together when you joined the health programme? It's to eat 5 servings of fruits and vegetables every day.<br><br>The following three fruits not only symbolize good fortune but can also help strengthen your immune system. Use the festive season as an opportunity to invest in better health and start the year full of energy!<br><br>Apples: Wishing You Peace and Safety All Year Long<br><br>In Chinese culture, the word for apple sounds similar to the word for peace.<br><br>Apples are rich in quercetin, a type of polyphenol often referred to as a "natural antihistamine" because it may help reduce allergic reactions.<br><br>The polyphenols in apples may also:<br>Help reduce visceral fat<br>Lower LDL ("bad") cholesterol<br>Support cardiovascular health<br>Oranges: Bringing Prosperity and Good Fortune<br><br>Oranges are associated with luck and prosperity during the Lunar New Year. They are rich in vitamin C, which acts as an antioxidant and may help inhibit the growth of cancer-causing agents.

		Step 3: Gradually exhale through your mouth while counting to 8.<br>Practice regularly whenever cravings arise.	hold a partial squat position to strengthen the core and lower body.<br><br>Even small amounts of activity can make a meaningful difference over time!	Vitamin C also:<br>Enhances iron absorption<br>Supports blood vessel health<br>May help reduce arterial stiffness<br><br>For individuals with high blood pressure, atherosclerosis, or coronary heart disease, oranges can be a particularly beneficial fruit choice.<br><br>Dragon Fruit: Symbolizing Prosperity and Vitality<br>Dragon fruit contains a wide range of nutrients, including: Plant proteins, Natural sugars, Carotenoids, Dietary fiber<br><br>Its fiber content supports digestion and may help the body eliminate waste products and toxins. Regular consumption may also contribute to healthier-looking skin and overall wellness.

25	Drinking too much alcohol harms your health! But how exactly?<br><br>Studies show that excessive drinking increases the risk of:<br><br>Cancer<br>Diabetes<br>Dementia and cognitive impairment<br>Stroke<br>Heart, liver, and kidney diseases<br><br>It also affects your sleep. Although some people find it easier to fall asleep after drinking, alcohol disrupts sleep patterns and prevents deep sleep. As a result, you may wake up feeling as though you hardly slept at all.<br><br>It weakens your immune system. With bacteria and viruses circulating widely, heavy drinking can make you more vulnerable to infectious diseases. Nobody wants that!	Smoking Affects More Than Just Your Breath<br><br>Many people know that the gut contains trillions of microorganisms, but did you know that the mouth also hosts a highly diverse microbiome? The oral microbiome is second only to the gut in size and diversity. When balanced, it helps digestion, supports immunity, and maintains oral health. Smoking disrupts this balance by altering the composition of oral bacteria. Some harmful bacteria increase in number and can travel to the digestive tract through saliva and swallowing. These bacteria may suppress beneficial gut microbes and create conditions that allow harmful microorganisms to thrive.<br><br>‼ Want a healthier digestive system? Quitting smoking can help! ‼<br>Reducing smoking may improve appetite and reduce gastrointestinal tension. Smoking stimulates the nervous system and can keep the digestive tract in a constant state of stress.<br>Smoking less may reduce heartburn and acid reflux symptoms. Smoking relaxes the lower esophageal sphincter, making stomach acid more likely to flow back into the esophagus.<br>Nicotine can impair the stomach's	Remember to Get 150 Minutes of Exercise Every Week!<br><br>Set higher expectations and healthier goals for the year ahead!<br><br>Research shows that both aerobic exercise and resistance training are beneficial for health. However, when it comes to sleep quality, resistance training appears to have greater benefits for:<br><br>Sleep duration<br>Sleep quality<br>Falling asleep more quickly<br><br>Recommended resistance training routine:<br><br>4 sets<br>10-13 repetitions per set<br>Weight equivalent to 50-80% of your maximum strength<br>Train 2-3 times per week<br><br>Stretching exercises can also promote relaxation and improve sleep, but sessions generally need to last at least 60 minutes to have similar effects.<br><br>Compared with long stretching sessions, a resistance-training workout of four sets may take less	Have you ever felt more tired or less able to concentrate after lunch because you skipped breakfast?<br>Breakfast is often called "the most important meal of the day," and for good reason. After an overnight fast, your body needs to replenish its energy stores to prepare for the day ahead. Research has shown that eating breakfast can improve memory and concentration, helping us perform at our best at work or school.<br><br>So, how can you create a nutritious breakfast?<br>Whole grains<br>Choose foods such as oatmeal or whole wheat toast. They are rich in fiber and help keep you feeling full for longer.<br>Protein<br>Options such as eggs, Greek yogurt, or tofu can provide sustained energy and support muscle maintenance and repair.<br>Fruits and vegetables<br>Fresh fruits and leafy green vegetables provide essential vitamins and minerals that help support a healthy immune system.<br>Healthy fats<br>Foods such as avocado and nuts can provide long-lasting energy, but should be consumed in moderation.<br>What have you been having for

		ability to neutralize acid. This allows stomach acid to damage the stomach lining.<br>Reducing smoking can lower the risk of gastric and duodenal ulcers while helping existing ulcers heal more quickly.	than 30 minutes, making it a more time-efficient option.	breakfast lately? Does it include some of the nutrients mentioned above? Why not spend the next week trying a different healthy breakfast each day? You could even start a "Breakfast Challenge" with friends or family and share your nutritious meal ideas with one another!<br>Adding a serving of fruit or vegetables to your breakfast is an easy way to work toward your goal of 5 daily servings of fruits and vegetables!

26	The first step to reducing alcohol consumption without added stress: stay hydrated!<br><br>Alcohol reduces the kidneys' ability to retain water, so it's important to replenish fluids during periods of cutting down or after drinking. Also, avoid drinking tea immediately after alcohol, as it may cause unmetabolized acetaldehyde to reach the kidneys too early.<br><br>Replace your usual "drinking ritual" with a new routine: choose sparkling water, alcohol-free beer, or mocktails during social occasions (or before alcoholic drinks) to satisfy the need to hold and sip a drink.	Encountering Obstacles on Your Smoking Cessation Journey?<br><br>These two smoking cessation services may be able to help!<br><br>Tung Wah Group of Hospitals Integrated Smoking Cessation Centre<br><br>The Mobile Smoking Cessation Clinic will continue to visit various locations across Hong Kong in May 2026.<br><br>This makes professional smoking cessation support more accessible and convenient.<br><br>Want to Quit Smoking Without Leaving Home?<br><br>The Department of Health's "Quit-at-Home" Programme offers a free smoking cessation medication delivery service, combined with professional telephone follow-up support to help you quit comfortably from home.<br><br>To register, call the Department of Health's Integrated Smoking Cessation Hotline:<br><br>1833 183<br><br>Professional support can significantly	No Time to Exercise and Frequently Suffering from Sciatic Pain?<br><br>Do you remember your health goal of achieving 150 minutes of exercise each week?<br><br>Kegel exercises involve contracting and relaxing the pelvic floor muscles to improve muscle control and strength.<br><br>In addition to benefiting urinary health, Kegel exercises can:<br><br>Enhance overall core stability<br>Improve blood circulation<br>Help relieve discomfort during exercise and daily activities<br>Support lower back and pelvic health<br>□<br>How to Perform Kegel Exercises<br><br>1) Lie on a flat surface, bend your knees, and place your feet flat on the floor. Rest your arms comfortably by your sides.<br>2) Gently raise your heels slightly and tighten your pelvic floor muscles, as if trying to stop urination. Avoid tightening your buttocks or thigh muscles.<br>3) Hold the contraction for 10 seconds, then slowly relax.<br>4) Rest for 10 seconds and repeat the	Have You Achieved Your Goal of 5 Servings of Fruits and Vegetables Today?<br><br>Eating plenty of fruits and vegetables not only provides essential vitamins and minerals, but also plays an important role in maintaining healthy bones.<br><br>Foods rich in: Calcium, Vitamin D, Vitamin K, Magnesium are especially important for bone health.<br>Examples include:<br>Dark green vegetables such as Spinach, Kale<br>Various fruits such as Oranges, Berries<br><br>These nutrients help:<br>Repair bone tissue<br>Maintain bone density<br>Reduce the risk of osteoporosis<br>Every serving of fruits and vegetables is an investment in your bone health.<br><br>Let's continue choosing a wide variety of fruits and vegetables and enjoy the energy and vitality they provide.<br>Remember, healthier eating today helps build stronger bones for the future.<br><br>Keep up the great work. You're laying a strong foundation for long-term health!

		improve your chances of successfully becoming smoke-free.	exercise 10-15 times. Remember to breathe normally throughout the exercise. Maintaining proper breathing also helps support cardiovascular fitness while you train your muscles.	
