# Supplementary material for: Effect of a digitally augmented general health promotion intervention on abstinence from health-risk behaviors among emergency department discharge patients: A randomized controlled trial
Source: PLoS Med. 2026 Sep 8;23(9):e1004916. doi: 10.1371/journal.pmed.1004916 (PMC13568513; doi:10.1371/journal.pmed.1004916)
Supplement: Supplementary file 4 [file pmed.1004916.s003.docx]

**S2 Text. Intervention, theoretical framework, participant flow, and outcomes**

This supplementary document provides structured details of the intervention, participant flow, and outcome measures to enhance transparency and reproducibility.

**Intervention description**

**Overview**

The intervention consisted of two components:
(1) a brief telephone‑based intervention delivered within three days of emergency department (ED) discharge using the AWARD model (Ask, Warn, Advise, Refer, Do‑it‑again); and
(2) standardized booster support delivered via weekly mobile instant messaging (WhatsApp or WeChat) for up to six months.
The intervention was designed as a pragmatic, low‑intensity health‑promotion strategy suitable for routine ED workflows.

**Brief telephone intervention**

The intervention was delivered by trained research nurses and lasted approximately 10 minutes. The AWARD steps comprised: assessment of current health‑risk behaviors; standardized warning on morbidity and mortality risks based on World Health Organization guidance; encouragement to abstain from a self‑selected behavior perceived as easiest to change; referral to relevant community or hotline services upon request; and re‑engagement using the same steps at follow‑up if abstinence was not achieved. The AWARD model served as a structured counseling framework, with delivery informed by behavioral theories described in the main manuscript.

**Booster messaging**

Participants in the intervention group received weekly, one‑way, non‑interactive WhatsApp or WeChat messages for up to six months. Messages were standardized, focused on reinforcing the selected behavior change, and provided brief reminders and encouragement. Participants received an average of approximately 24 messages.

**Control condition**

Control participants received a single brief telephone call within three days of ED discharge, including basic advice on health‑risk behaviors and reference to a standardized healthy lifestyle leaflet. No structured counseling, individualized goal setting, or booster messaging was provided.

**Conceptual and theoretical framework**

The intervention was informed by a selective integration of three complementary frameworks: the Theory of Planned Behavior, the foot‑in‑the‑door technique, and self‑determination theory. Rather than applying all constructs from each framework, we adopted a pragmatic, parsimonious approach, drawing on specific theoretical elements relevant to a brief emergency‑department–initiated intervention. These frameworks were used to guide intervention design and sequencing, rather than to test theory‑specific mechanisms.

**Theory of planned behavior**

According to this theory, an individual’s intention to engage in a health-related behavior is shaped by three proximal determinants: attitudes, subjective norms, and perceived behavioral control. Attitudes reflect a person’s evaluation of the behavior, including beliefs about its likelihood and perceived benefits or consequences. Subjective norms refer to perceived social expectations or pressures regarding whether the behavior should be performed, while perceived behavioral control captures an individual’s sense of autonomy and confidence in their ability to carry out the behavior. Together, these factors influence behavioral intentions, which are considered the most immediate predictors of actual behavior.

**Foot-in-the-door technique**

The foot-in-the-door technique, first introduced by Freeman and Fraser, posits that individuals who agree to an initial small, simple request are more likely to comply with a subsequent larger request. Initial compliance fosters confidence and reinforces self-perceptions of capability and willingness, thereby increasing the likelihood of further engagement. In the context of this intervention, this technique can be leveraged to facilitate participant recruitment and enhance adherence to program components.

**Self-determination theory**

Self-determination theory highlights the importance of intrinsic motivation and autonomy in sustaining behavioral change. Autonomy fosters self-efficacy and competence, both of which are important for long-term success. Allowing individuals to choose and abstain from a health-risk behavior that they perceive as the easiest behavior to change is expected to increase their willingness to participate in an intervention (resulting in a higher response rate) and maintain engagement throughout the study (leading to a higher retention rate). Fostering autonomy and competence is primarily expected to enhance the likelihood of successfully abstaining from a health-risk behavior.

Our intervention was designed to first influence participants’ attitudes and subjective norms through risk communication. Subsequent incorporation of the foot-in-the-door technique along with the principles of self-determination theory was anticipated to the effectiveness of the intervention at increasing the participants’ willingness to abstain from health-risk behaviors.

Each framework informed a distinct phase of the intervention. The Theory of Planned Behavior guided the initial motivational component by shaping risk communication aimed at influencing attitudes and perceived behavioral control. Self‑determination theory informed the emphasis on autonomy and competence, operationalized through participant choice of target behavior and supportive follow‑up messaging. The foot‑in‑the‑door technique informed the sequential behavior‑change strategy, whereby participants were encouraged to begin with a self‑selected, easier behavior before progressing to additional health‑risk behaviors.

**Participant flow**

Participants were screened in the ED prior to discharge by emergency nurses and referred to a research assistant for informed consent and baseline assessment. Randomization occurred after baseline data entry using a computer‑generated, stratified block randomization sequence. Participant flow through screening, enrollment, allocation, follow‑up, and analysis is summarized in the CONSORT flow diagram (Figure 1). All randomized participants were included in the intention‑to‑treat analysis, with follow‑up assessments conducted at 3, 6, and 12 months via telephone.

**Outcome measures**

**Primary outcome**

Abstinence from at least one health‑risk behavior at 6 months.

**Secondary outcomes**

Abstinence from at least one health‑risk behavior at 12 months and reduction in the total number of health‑risk behaviors at 6 and 12 months.

**Definitions and assessment**

Health‑risk behaviors and criteria for successful abstinence were defined as a priori using established public health benchmarks (Table 1). Outcomes were assessed by research nurses not involved in intervention delivery, following a standardized script. All outcomes were self‑reported. Missing outcome data were handled conservatively in the intention‑to‑treat analysis by assuming no change from baseline.

**Reproducibility considerations**

Reproducibility was supported by standardized intervention scripts and messages, clear differentiation between intervention and control conditions, pre‑specified outcome definitions and assessment time points, and transparent reporting of participant flow and analytical methods.
