# Supplementary material for: Effect of a digitally augmented general health promotion intervention on abstinence from health-risk behaviors among emergency department discharge patients: A randomized controlled trial
Source: PLoS Med. 2026 Sep 8;23(9):e1004916. doi: 10.1371/journal.pmed.1004916 (PMC13568513; doi:10.1371/journal.pmed.1004916)
Supplement: Supplementary file 5 [file pmed.1004916.s005.docx]

Table S1. Criteria for determining each health-risk behavior and for defining successful abstinence from these behaviors

| **Health-risk Behaviors** | **Criteria for Determining Each Health-Risk Behavior** | **Criteria for Defining Successful Abstinence from Each Health-Risk Behavior** |
| --- | --- | --- |
| Tobacco use | Smoked at least 1 cigarette a day over the past 30 days | Self-reported 7-day point prevalence of smoking abstinence |
| Binge drinking | Reporting ≥1 binge drinking episodes (consumption of five or more alcoholic beverages on one occasion) in the past month. | Self-report no binge drinking episode (consumption of five or more alcoholic beverages on one occasion) in the past month. |
| Unhealthy diet | Have consumed less than five servings of fruit and vegetables per day or have had a daily intake of less than 400 grams of fruit and vegetables | Have consumed at least five servings of fruit and vegetables per day or have had a daily intake of at least 400 grams of fruit and vegetables |
| Physical inactivity | (1) Performed less than 150 minutes of moderate-intensity aerobic physical activity throughout the week, or (2) performed less than 75 minutes of vigorous-intensity aerobic physical activity throughout the week | Performed at least 150 minutes of moderate-intensity aerobic physical activity throughout the week, or performed at least 75 minutes of vigorous-intensity aerobic physical activity throughout the week |
