# Supplementary material for: Effect of a digitally augmented general health promotion intervention on abstinence from health-risk behaviors among emergency department discharge patients: A randomized controlled trial
Source: PLoS Med. 2026 Sep 8;23(9):e1004916. doi: 10.1371/journal.pmed.1004916 (PMC13568513; doi:10.1371/journal.pmed.1004916)
Supplement: Supplementary file 6 [file pmed.1004916.s006.docx]

Table S2. Generalised equation model for primary and secondary outcomes at 6- and 12-month follow-up among 572 participants

|  | RR (95% CI) ^a^ | | aRR (95% CI) ^a, b^ | |
| --- | --- | --- | --- | --- |
|  | Control | Intervention | Control | Intervention |
| **Primary outcome** |  |  |  |  |
| Abstinence of ≥1 health-risk behaviour at 6 months | 1 | 1.51 (1.13-2.02) ^**^ | 1 | 1.53 (1.15-2.04) ^**^ |
| **Secondary outcomes** |  |  |  |  |
| Abstinence of ≥1 health-risk behaviour at 12 months | 1 | 1.24 (0.89-1.75) | 1 | 1.19 (0.85-1.67) |
| Having reduced the total number of health-risk behaviours | | | | |
| 6 months | 1 | 1.54 (1.11-2.15) ^*^ | 1 | 1.51 (1.09-2.10) ^*^ |
| 12 months | 1 | 1.48 (1.07-2.05) ^*^ | 1 | 1.44 (1.05-1.99) ^*^ |

^a^ derived from a generalized estimating equation model with a binomial log link function using robust standard errors clustered by participant; ^b^ adjusted for age, sex, education level, and baseline smoking status, alcohol use, diet and physical activity. ^*^ p<0.05; ^**^ p<0.01; ^***^ p<0.001
