# Supplementary material for: Effect of a digitally augmented general health promotion intervention on abstinence from health-risk behaviors among emergency department discharge patients: A randomized controlled trial
Source: PLoS Med. 2026 Sep 8;23(9):e1004916. doi: 10.1371/journal.pmed.1004916 (PMC13568513; doi:10.1371/journal.pmed.1004916)
Supplement: Supplementary file 7 [file pmed.1004916.s007.docx]

Table S3. Generalised equation model for primary outcomes at 6- and 12-month follow-up (Complete case analysis)

|  | RR (95% CI) ^a^ | | aRR (95% CI) ^a, b^ | |
| --- | --- | --- | --- | --- |
|  | Control | Intervention | Control | Intervention |
| **Primary outcome** |  |  |  |  |
| Abstinence of ≥1 health-risk behaviour at 6 months | 1 | 1.31 (1.01-1.70) ^*^ | 1 | 1.30 (1.01-1.67) ^*^ |
| **Secondary outcomes** |  |  |  |  |
| Abstinence of ≥1 health-risk behaviour at 12 months | 1 | 1.07 (0.79-1.44) | 1 | 1.03 (0.76-1.40) |
| Having reduced the total number of health-risk behaviours |  |  |  |  |
| 6 months |  | 1.34 (0.99-1.82) | 1 | 1.26 (0.94-1.69) |
| 12 months |  | 1.27 (0.88-1.83) | 1 | 1.24 (0.87-1.79) ^c^ |

**^a^** Derived from a generalized estimating equation model with a binomial log-link function and robust standard errors clustered by participant;
**^b^** Adjusted for age, sex, education level, baseline smoking status, alcohol use, diet, and physical activity;
**^c^** Derived from a generalized estimating equation model with a modified Poisson regression and robust standard errors clustered by participant.
**P* < 0.05; ****P** < 0.01; ******P*** < 0.001.
