# Supplementary material for: Effect of a digitally augmented general health promotion intervention on abstinence from health-risk behaviors among emergency department discharge patients: A randomized controlled trial
Source: PLoS Med. 2026 Sep 8;23(9):e1004916. doi: 10.1371/journal.pmed.1004916 (PMC13568513; doi:10.1371/journal.pmed.1004916)
Supplement: Supplementary file 8 [file pmed.1004916.s008.docx]

Table S4. Generalised equation model for primary outcome among 572 participants (multiple imputation)

|  | RR (95% CI) ^a^ | | aRR (95% CI) ^a, b^ | |
| --- | --- | --- | --- | --- |
|  | Control | Intervention | Control | Intervention |
| Abstinence of ≥1 health-risk behaviour at 6 months | 1 | 1.31 (1.04-1.67) ^*^ | 1 | 1.32 (1.05-1.66) ^*^ |

^a^ derived from a generalized estimating equation model using a modified Poisson regression with robust standard errors clustered by participant; ^b^ adjusted for age, sex, education level, and baseline smoking status, alcohol use, diet and physical activity. ^*^ p<0.05; ^**^ p<0.01; ^***^ p<0.001
